# Genetic studies of abdominal MRI data identify genes regulating hepcidin as major determinants of liver iron concentration

**DOI:** 10.1016/j.jhep.2019.05.032

**Published:** 2019-09

**Authors:** Henry R. Wilman, Constantinos A. Parisinos, Naeimeh Atabaki-Pasdar, Matt Kelly, E. Louise Thomas, Stefan Neubauer, IMI DIRECT Consortium, Christopher Jennison, Beate Ehrhardt, Patrick Baum, Corinna Schoelsch, Jan Freijer, Rolf Grempler, Ulrike Graefe-Mody, Anita Hennige, Christiane Dings, Thorsten Lehr, Nina Scherer, Iryna Sihinecich, Francois Pattou, Violeta Raverdi, Robert Caiazzo, Fanelly Torres, Helene Verkindt, Andrea Mari, Andrea Tura, Toni Giorgino, Roberto Bizzotto, Philippe Froguel, Amelie Bonneford, Mickael Canouil, Veronique Dhennin, Caroline Brorsson, Soren Brunak, Federico De Masi, Valborg Gudmundsdóttir, Helle Pedersen, Karina Banasik, Cecilia Thomas, Peter Sackett, Hans-Henrik Staerfeldt, Agnete Lundgaard, Birgitte Nilsson, Agnes Nielsen, Gianluca Mazzoni, Tugce Karaderi, Simon Rasmussen, Joachim Johansen, Rosa Allesøe, Andreas Fritsche, Barbara Thorand, Jurek Adamski, Harald Grallert, Mark Haid, Sapna Sharma, Martina Troll, Jonathan Adam, Jorge Ferrer, Heather Eriksen, Gary Frost, Ragna Haussler, Mun-gwan Hong, Jochen Schwenk, Mathias Uhlen, Claudia Nicolay, Imre Pavo, Birgit Steckel-Hamann, Melissa Thomas, Kofi Adragni, Han Wu, Leen't Hart, Slieker Roderick, Nienke van Leeuwen, Koen Dekkers, Francesca Frau, Johann Gassenhuber, Bernd Jablonka, Petra Musholt, Hartmut Ruetten, Joachim Tillner, Tania Baltauss, Oana Bernard Poenaru, Nathalie de Preville, Marianne Rodriquez, Manimozhiyan Arumugam, Kristine Allin, Line Engelbrechtsen, Torben Hansen, Tue Hansen, Annemette Forman, Anna Jonsson, Oluf Pedersen, Avirup Dutta, Josef Vogt, Henrik Vestergaard, Markku Laakso, Tarja Kokkola, Teemu Kuulasmaa, Paul Franks, Nick Giordano, Hugo Pomares-Millan, Hugo Fitipaldi, Pascal Mutie, Maria Klintenberg, Margit Bergstrom, Leif Groop, Martin Ridderstrale, Naeimeh Atabaki Pasdar, Harshal Deshmukh, Alison Heggie, Dianne Wake, Donna McEvoy, Ian McVittie, Mark Walker, Andrew Hattersley, Anita Hill, Angus Jones, Timothy McDonald, Mandy Perry, Rachel Nice, Michelle Hudson, Claire Thorne, Emmanouil Dermitzakis, Ana Viñuela, Louise Cabrelli, Heather Loftus, Adem Dawed, Louise Donnelly, Ian Forgie, Ewan Pearson, Colin Palmer, Andrew Brown, Robert Koivula, Agata Wesolowska-Andersen, Moustafa Abdalla, Nicky McRobert, Juan Fernandez, Yunlong Jiao, Neil Robertson, Stephen Gough, Jane Kaye, Miranda Mourby, Anubha Mahajan, Mark McCarthy, Nisha Shah, Harriet Teare, Reinhard Holl, Anitra Koopman, Femke Rutters, Joline Beulens, Lenka Groeneveld, Anitra Koopman, Jimmy Bell, Louise Thomas, Brandon Whitcher, Anubha Mahajan, Aroon D. Hingorani, Riyaz S. Patel, Harry Hemingway, Paul W. Franks, Jimmy D. Bell, Rajarshi Banerjee, Hanieh Yaghootkar

**Affiliations:** 1 Department of Mathematical Sciences, University of Bath, Bath, UK; 2 Boehringer Ingelheim Pharma GmbH & Co. KG, Translational Medicine & Clinical Pharmacology, Birkendorferstr.65, 88397 Biberach an der Riss, Germany; 3 Boehringer Ingelheim Pharma GmbH & Co. KG, Medicine Therapeutic Area Metabolism 1, Binger Strasse 173, 55216 Ingelheim am Rhein, Germany; 4 Boehringer Ingelheim Pharma GmbH & Co. KG, Medicine Therapeutic Area Metabolism 1, Birkendorferstr.65, 88397 Biberach an der Riss, Germany; 5 Clinical Pharmacy, Saarland University, Campus C2.2, 66123 Saarbrücken, Germany; 6 Centre Hospitalier Régional Universitaire de Lille 2, Av Oscar Lambret, 59037 Lille Cedex, France; 7 Institute of Neuroscience, National Research Council, Corso Stati Uniti 4, 35127 Padova, Italy; 8 Centre National de la Recherche Scientific CNRS, Délégation Nord Pas-de-Calais et Picardie du CNRS Espace Recherche et Innovation, 2, rue des Canonniers, 59046 Lille Cedex, France; 9 Imperial College London, Department of Genomics of Common Disease, School Of Public Health, Hammersmith Hospital Campus, Imperial College Faculty of Medicine, Burlington-Danes Building, Du Cane Road, London W12 0NN, UK; 10 Center for Biological Sequence Analysis, Dept. of Systems Biology, Technical University of Denmark, Kongens Lyngby, Denmark; 11 Novo Nordisk Foundation Center for Protein Research, Faculty of Health and Medical Sciences, University of Copenhagen, Blegdamsvej 3A, DK-2200 Copenhagen, Denmark; 12 Wellcome Trust Centre for Human Genetics, University of Oxford, Oxford OX3 7BN, UK; 13 Novo Nordisk Foundation Center for Protein Research, Translational Disease Systems Biology, University of Copenhagen, Blegdamsvej 3A, DK-2200 Copenhagen, Denmark; 14 Medizinische Universitätsklinik Tübingen, Eberhard Karls Universität Tübingen, Otfried Müller Straße 10, 72076 Tübingen, Germany; 15 Institute of Epidemiology II, Research Unit of Diabetes Epidemiology, Helmholtz Zentrum München Research Center for Environmental Health, Ingolstaedter Landstr. 1, 85764 Neuherberg, Germany; 16 German Center for Diabetes Research (DZD), Neuherberg, Germany; 17 Helmholtz Zentrum München, Ingolstaedter Landstr. 1, 85764 Neuherberg, Germany; 18 Institute of Experimental Genetics, Genome Analysis Center, Ingolstaedter Landstr. 1, 85764 Neuherberg, Germany; 19 German Center for Diabetes Research (DZD), 85764 München-Neuherberg, Germany; 20 Institute of Epidemiology II, Research Unit of Molecular Epidemiology, Helmholtz Zentrum München Research Center for Environmental Health, Neuherberg, Germany; 21 Clinical Cooperation Group Type 2 Diabetes, Helmholtz Zentrum München, Neuherberg, Germany; 22 Clinical Cooperation Group Nutrigenomics and Type 2 Diabetes, Helmholtz Zentrum Munchen, German Research Center for Environmental Health, 85764 Neuherberg, Germany; 23 Technische Universität München, 85350 Freising-Weihenstephan, Germany; 24 Institut d'Investigacions Biomediques August Pi i Sunye, Centre Esther Koplowitz, c/Rosselló 153, Barcelona 08036, Spain; 25 Nutrition and Dietetics Research Group, Imperial College London SW7 2AZ, UK; 26 Affinity Proteomics, Science for Life Laboratory, School of Biotechnology, KTH – Royal Institute of Technology, Box 1031, SE-171 21 Solna, Sweden; 27 Lilly Deutschland GmbH, Werner-Reimers-Str. 2-4, 61352 Bad Homburg, Germany; 28 Eli Lilly Regional Operations Ges.m.b.H., Koelblgasse 8-10, 1030 Vienna, Austria; 29 Eli Lilly Regional Operations Ges.m.b.H., Koelblgasse 8-10, 1030 Vienna, Austria; 30 Lilly Research Laboratories, Eli Lilly and Company, Indianapolis, IN 46285, USA; 31 Leiden University Medical Center, Dept. of Molecular Cell Biology, Albinusdreef 2, 2333ZA Leiden, The Netherlands; 32 Leiden University Medical Center, Dept. of Molecular Epidemiology, Albinusdreef 2, 2333ZA Leiden, The Netherlands; 33 Diabetes Division, Sanofi-Aventis Deutschland GmbH, 65926 Frankfurt am Main, Germany; 34 Strategy & Innovation, Sanofi-Aventis Deutschland GmbH, 65926 Frankfurt am Main, Germany; 35 Clinical Operations, Sanofi-Aventis Deutschland GmbH, 65926 Frankfurt am Main, Germany; 36 Translational & Clinical Research, Metabolism Innovation Pole, Institut de Recherches Internationales Servier, 92284 Suresnes Cedex, France; 37 Biotech&Biomarkers Research Department, Institut de Recherches Internationales Servier, 78290 Croissy sur Seine, France; 38 The Novo Nordisk Center for Basic Metabolic Research, Section of Metabolic Genetics, Faculty of Health and Medical Science, University of Copenhagen, Copenhagen DK-2100, Denmark; 39 The Novo Nordisk Center for Basic Metabolic Research, Section of Metabolic Genetics, Faculty of Health and Medical Science, University of Copenhagen, Copenhagen DK-2101, Denmark; 40 The Novo Nordisk Center for Basic Metabolic Research, Section of Metabolic Genetics, Faculty of Health and Medical Science, University of Copenhagen, Copenhagen DK-2101, Denmark; 41 The Novo Nordisk Center for Basic Metabolic Research, Section of Metabolic Genetics, Faculty of Health and Medical Science, University of Copenhagen, Copenhagen DK-2101, Denmark; 42 Institute of Clinical Medicine, Internal Medicine, University of Eastern Finland, 70210 Kuopio, Finland; 43 Genetic and Molecular Epidemiology, Department of Clinical Science, Lund University, Skåne University Hospital Malmö, CRC, 91-10, 205 02 Malmö, Sweden; 44 VO Endokrinologi, Enheten för diabetesstudier Lasarettsgatan 15, Hudhuset plan 3 Skånes Universitetssjukhus i Lund, Sweden; 45 Department of Clinical Sciences, Diabetes & Endocrinology Unit, Lund University, Skåne University Hospital Malmö, CRC, 91-12, 205 02 Malmö, Sweden; 46 Institute of Cellular Medicine, Faculty of Medical Sciences, Newcastle University, UK; 47 Diabetes Research Network, Royal Victoria Infirmary, Newcastle upon Tyne, UK; 48 NIHR Exeter Clinical Research Facility, University of Exeter Medical School, Exeter EX25DW, UK; 49 Blood Sciences, Royal Devon and Exeter NHS Foundation Trust, Barrck Rd, Wonford, Exeter EX2 5DW, UK; 50 NIHR Exeter Clinical Research Facility, Royal Devon and Exeter NHS Foundation Trust, Barrck Rd, Wonford, Exeter EX2 5DW, UK; 51 Department of Genetic Medicine and Development, University of Geneva Medical School, 1211 Geneva, Switzerland; 52 Clinical Research Centre, Ninewells Hospital and Medical School, University of Dundee, Dundee, Scotland DD1 9SY, UK; 53 Molecular and Clinical Medicine, Ninewells Hospital and Medical School, University of Dundee, Dundee, Scotland DD1 9SY, UK; 54 Oxford Centre for Diabetes Endocrinology and Metabolism, University of Oxford, Oxford OX3 7LJ, UK; 55 Oxford Centre for Diabetes Endocrinology and Metabolism, University of Oxford, Oxford OX3 7LE, UK; 56 Nuffield Department of Population Health, Centre for Health, Law and Emerging Technologies (HeLEX), University of Oxford, Oxford OX2 7DD, UK; 57 University of Ulm, Institute for Epidemiology and medical Biometry, ZIBMT, Albert-Einstein-Allee 41, D-89081 Ulm, Germany; 58 Department of Epidemiology and Biostatistics, VUMC, de Boelelaan 1089a, 1081 HV Amsterdam, The Netherlands; 59 Research Centre for Optimal Health, Deparment of Life Sciences, University of Westminster, London, UK; 1 Research Centre for Optimal Health, School of Life Sciences, University of Westminster, London, UK; 2 Perspectum Diagnostics Ltd., Oxford, UK; 3 Institute of Health Informatics, Faculty of Population Health Sciences, University College London, London, UK; 4 Department of Clinical Sciences, Genetic and Molecular Epidemiology Unit, Lund University, Skåne University Hospital Malmö, Malmö, Sweden; 5 Oxford Centre for Clinical Magnetic Resonance Research, Division of Cardiovascular Medicine, Oxford NIHR Biomedical Research Centre, University of Oxford, Oxford, UK; 6 Wellcome Centre for Human Genetics, University of Oxford, Oxford, UK; 7 Institute of Cardiovascular Science, Faculty of Population Health Sciences, University College London, London, UK; 8 Genetics of Complex Traits, College of Medicine and Health, University of Exeter, Exeter, UK

**Keywords:** Magnetic resonance imaging, Iron, Metabolism, Metabolic syndrome, Genome-wide association study, Genetics

## Abstract

**Background & Aims:**

Excess liver iron content is common and is linked to the risk of hepatic and extrahepatic diseases. We aimed to identify genetic variants influencing liver iron content and use genetics to understand its link to other traits and diseases.

**Methods:**

First, we performed a genome-wide association study (GWAS) in 8,289 individuals from UK Biobank, whose liver iron level had been quantified by magnetic resonance imaging, before validating our findings in an independent cohort (n = 1,513 from IMI DIRECT). Second, we used Mendelian randomisation to test the causal effects of 25 predominantly metabolic traits on liver iron content. Third, we tested phenome-wide associations between liver iron variants and 770 traits and disease outcomes.

**Results:**

We identified 3 independent genetic variants (rs1800562 [C282Y] and rs1799945 [H63D] in *HFE* and rs855791 [V736A] in *TMPRSS6*) associated with liver iron content that reached the GWAS significance threshold (*p* <5 × 10^−8^). The 2 *HFE* variants account for ∼85% of all cases of hereditary haemochromatosis. Mendelian randomisation analysis provided evidence that higher central obesity plays a causal role in increased liver iron content. Phenome-wide association analysis demonstrated shared aetiopathogenic mechanisms for elevated liver iron, high blood pressure, cirrhosis, malignancies, neuropsychiatric and rheumatological conditions, while also highlighting inverse associations with anaemias, lipidaemias and ischaemic heart disease.

**Conclusion:**

Our study provides genetic evidence that mechanisms underlying higher liver iron content are likely systemic rather than organ specific, that higher central obesity is causally associated with higher liver iron, and that liver iron shares common aetiology with multiple metabolic and non-metabolic diseases.

**Lay summary:**

Excess liver iron content is common and is associated with liver diseases and metabolic diseases including diabetes, high blood pressure, and heart disease. We identified 3 genetic variants that are linked to an increased risk of developing higher liver iron content. We show that the same genetic variants are linked to higher risk of many diseases, but they may also be associated with some health advantages. Finally, we use genetic variants associated with waist-to-hip ratio as a tool to show that central obesity is causally associated with increased liver iron content.

## Highlights

• Variants in *HFE* and *TMPRSS6* are associated with higher liver iron.
• There is genetic evidence that higher central obesity causes higher liver iron.
• Liver iron variants are not organ specific and associate with multiple diseases.

## Graphical abstract

**Figure:**
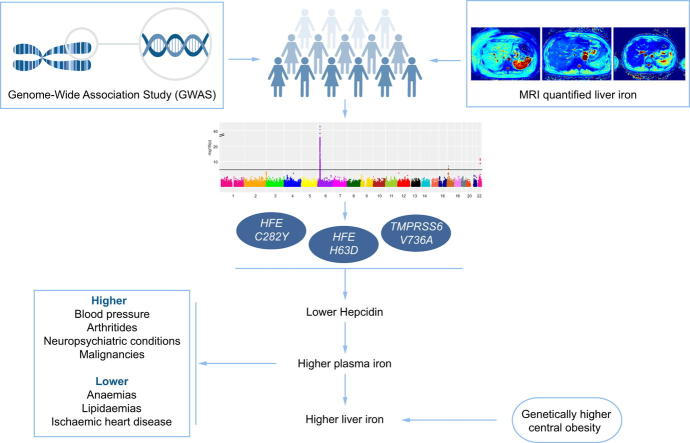

## Introduction

Liver disease constitutes the third most common cause of premature death in the UK, and its prevalence is substantially higher compared to other countries in Western Europe.[1], [2], [3] Excess liver iron is associated with increased severity and progression of liver diseases including cirrhosis and hepatocellular carcinoma in individuals with non-alcoholic fatty liver disease (NAFLD),[4], [5], [6] and is the direct cause of liver disease in those with hereditary haemochromatosis and thalassaemia.[7], [8] Observational associations have been described between excess liver iron content and several metabolic diseases such as high blood pressure, obesity, polycystic ovarian syndrome and type 2 diabetes, in a condition recognised as dysmetabolic iron overload syndrome (DIOS) which affects up to 5–10% of the general population.[9], [10]

The associations between excess liver iron and hepatic and non-hepatic diseases necessitate exploration of underlying pathophysiological mechanisms. Studies of patients with hereditary haemochromatosis, with autosomal recessive mutations, show they have higher liver iron, measured from biopsies, when compared to controls. However, no studies have been performed in unselected populations. Furthermore, it is unknown whether iron accumulation is a systemic disorder involving multiple organs or whether there are mechanisms specific to the liver. Previous genome-wide association studies (GWAS) have focussed on peripheral biochemical markers of iron status that do not correlate well with liver iron.^11^

Measuring liver iron has traditionally been difficult. Liver biopsy, the “gold standard” for assessment of liver iron, is an invasive procedure and therefore unsuitable for population research studies. An alternative is magnetic resonance imaging (MRI); a non-invasive, quick, robust and validated method for quantifying liver iron content.^12^ The availability of genetic and clinical data, as well as MRI scans of livers in the UK Biobank cohort has provided an unparalleled opportunity to study the genetics of liver iron content in a population-based cohort.

**Fig. 1:**
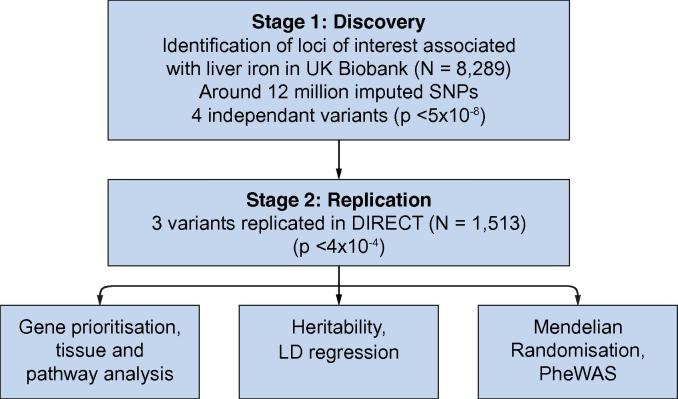
**Study design.** GWAS on liver iron content was performed in UK Biobank (N = 8,289) and replicated in IMI DIRECT (N = 1,115). GWAS, genome-wide association study; SNPs, single-nucleotide polymorphisms.

The aim of this study was to (i) identify genetic variants specifically associated with liver iron content, (ii) investigate which metabolic traits and diseases might cause higher liver iron content, and (iii) characterise the traits/diseases associated with liver iron content susceptibility variants. To facilitate this, we performed the first GWAS of MRI-determined liver iron content in 8,289 UK Biobank participants and replicated our findings in an independent cohort of 1,513 participants of European ancestry from the IMI DIRECT study (Fig. 1).[13], [14]

## Patients and methods

### UK Biobank participants

The UK Biobank consists of over 500,000 individuals aged 37–73 years (99.5% were between 40 and 69 years of age) who were recruited between 2006 and 2010 from across the UK.^13^ This research has been conducted using the data obtained via UK Biobank Access Application number 9914. UK Biobank field numbers used for this analysis can be found in Table S1. We used data from the first subset of UK Biobank participants invited for multiparametric MRI imaging between 2014 and 2016.^15^ After image analysis and quality control steps (see below), liver iron was available for 8,674 individuals who also had genetic data. We based our study on 8,289 individuals of white European descent as defined by principal component (PC) analysis.

### Genetic data

Protocols for the participant genotyping, data collection, and quality control have previously been described in detail.^13^ Briefly, participants were genotyped using 1 of 2 purpose-designed arrays (UK BiLEVE Axiom Array (n = 50,520) and UK Biobank Axiom Array (n = 438,692)) with 95% marker overlap. We excluded individuals who were identified by the UK Biobank as outliers based on either genotyping missingness rate or heterogeneity, or whose sex inferred from the genotypes did not match their self-reported sex. We removed individuals with a missingness >5% across variants which passed our quality control procedure. We used the latest release which included imputed data using 2 reference panels: a combined UK10K and 1000 Genomes panel and the Haplotype Reference Consortium panel. We limited our analysis to genetic variants with a minimum minor allele frequency (MAF) >1% and imputation quality score (INFO) >0.3.

### Imaging protocol and analysis

The imaging protocol and analysis of liver iron content in UK Biobank participants has previously been published.^15^ Briefly, participants were scanned at the UK Biobank centre in Cheadle (UK) using a Siemens 1.5 T Magnetom Aera. A single-breath-hold MRI sequence was acquired as a single transverse slice captured through the centre of the liver, superior to the porta hepatis. This sequence forms part of the UK Biobank abdominal imaging protocol. The data were analysed using the Liver*MultiScan™* Discover software (version 4.0; Perspectum Diagnostics Ltd, UK) by a team of trained analysts, blinded to any subject variables. Analysts selected three 15 mm diameter circular regions of interests, to cover a representative sample of the liver parenchyma, avoiding vessels, bile ducts and other organs. The repeatability and reproducibility of the image analysis was high.^15^

### Genome-wide association analysis

We performed the association tests using 2 different software: (1) GEMMA version 0.96 as our main analysis using all individuals of genetically defined Europeans (n = 8,289),^16^ and (2) PLINK version 1.9 as our sensitivity analysis using unrelated white British individuals (defined in UK Biobank field 22,006, n = 6,758).^17^

GEMMA applies a linear mixed-model to adjust for the effects of population structure and relatedness. Therefore, we increased our power by including all related individuals of European descent. The relatedness matrix was computed using common (MAF >5%) genotyped variants that passed quality control. Prior to association testing, liver iron was first log-transformed and then adjusted for age, sex and study centre, as well as body mass index (BMI) or alcohol consumption in our sensitivity analysis. We then performed inverse normal transformation on the values. At runtime, we included genotyping array (as a categorical variable for UKBileve array, UKB Axiom array interim release and UKB Axiom array full release) as a covariate.

We used PLINK to perform a sensitivity analysis. Prior to association testing, liver iron was adjusted for age, sex, BMI and genotyping array, and then we quantile normalised the resulting values. At runtime, we included the first 10 genetic PCs (UK Biobank field 22,009) as covariates to control for confounding by population stratification. We used Quanto (http://biostats.usc.edu/Quanto.html) to calculate our discovery GWAS power in 8,289 individuals from the UK Biobank at different allele frequencies and effect sizes at α level 5 × 10^−8^ assuming additive effect model (Fig. S1).

### Linkage disequilibrium score regression and cross-trait genetic correlation analysis

We used LDHub to conduct linkage disequilibrium (LD) score regression and heritability analysis. LD Hub is a centralized database of summary level GWAS for >100 diseases/traits from publicly available resources/consortia and uses a web interface that automates LD score regression, heritability and cross-trait genetic correlation analysis pipeline.^18^ We ran heritability analysis as well as genetic correlation analysis across 448 potentially relevant traits. SNP-based heritability (*h*^2^_SNP_) is the proportion of total variation in liver iron content due to the additive genetic variation between individuals in our study population.

### Gene-set and tissue expression enrichment analysis

We performed gene-set and tissue expression analyses using MAGMA.^19^ Lead variants were assigned to a minimum *p* value of 5 × 10^−8^. We used the default settings provided by the software. We chose 1000 Genomes Phase 3 as the reference panel population. The minimum minor allele frequency was set to 1%. We used a maximum allowed distance of 250 kb between LD blocks for variants to be included in the same locus.

For gene-set enrichment analyses, positional mapping was used with variants assigned to a gene if they were within the gene start and end points (by setting the distance either side to 0 kb). Only protein-coding genes were included in the mapping process. Tested gene sets include BioCarta, REACTOME, KEGG and GO. Bonferroni correction was used to adjust for the number of gene sets tested. Analysis of differentially expressed genes was based on data from GTEx v6 RNA-seq data.^20^

### Replication analysis

Associations reaching *p* <5 × 10^−8^ were followed up in the IMI DIRECT cohort. IMI DIRECT includes 1,513 participants who had both liver iron and GWAS data to replicate our findings. The IMI DIRECT consortium is a collaboration among investigators from a range of European academic institutions and pharmaceutical companies.^14^ Liver iron was measured using a T2*-based multi-echo MRI technique.^21^ DNA extraction was carried out using Maxwell 16 Blood DNA purification kits and a Maxwell 16 semi-automated nucleic acid purification system (Promega). Genotyping was conducted using the Illumina HumanCore array (HCE24 v1.0) and genotypes were called using Illumina’s GenCall algorithm. A total of 517,958 markers passed quality control procedures. We took autosomal variants with MAF >1% that passed quality control and constructed axes of genetic variation using PC analysis implemented in the GCTA software to identify ethnic outliers defined as non-European ancestry using the 1000 Genomes samples as reference. We identified 6 individuals as ethnic outliers.

We performed the association tests in 3 models: (i) non-diabetic participants (n = 1,010), (ii) diabetic participants (n = 503), and (iii) combined (n = 1,513). We took residuals from a model of liver iron and age, sex, BMI, 10 PCs and centres, before performing inverse normal transformation on the values.

### Sensitivity analyses

We performed 4 sensitivity analyses. First, to assess whether there is any sex-specific association, we carried out GWASs in men and women separately. Second, to test whether relatedness was responsible for any of the individual variant associations and replicate GEMMA results, we ran a GWAS using only unrelated Europeans individuals in the UK Biobank and a different GWAS software tool (PLINK version 1.9). Third, we adjusted models for alcohol intake frequency (field 1,558; categories treated as ordinal scale – “Never” = 0 to “Daily or almost daily” = 5) measured at baseline that may have had an impact on liver iron. Individuals responding “Do not know” or “Prefer not to answer” for “Alcohol intake frequency” were excluded from this sensitivity analysis. Fourth, to investigate the potential for collider bias resulting from conditioning liver iron on BMI, we performed a GWAS of liver iron without adjustment for BMI.

### Mendelian randomisation

Multiple traits have shown association with liver iron in observational studies, including BMI, lipids and non-alcoholic fatty liver disease.[10], [22], [23], [24] We therefore investigated the causal effects of 25 predominantly metabolic traits using 2-sample Mendelian randomisation analysis.^25^ Mendelian randomisation is a method that uses genetic variants associated with the exposure (*e.g.* metabolic traits) to infer causal relationships between an exposure and an outcome (*e.g.* liver iron content). This method relies on a simple principle; if a modifiable exposure is causal for a disease, then the genetic variants associated with that exposure will also be associated with disease risk. Since genetic variants are inherited at birth, Mendelian randomisation experiments are free from confounding and biases that are seen in observational studies.

Following correction for multiple testing, associations with a false discovery rate (FDR) <5% were considered statistically significant. We used the inverse variance weighted approach (IVW) as our main analysis, and MR-Egger and penalised weighted median as sensitivity analyses in the event of unidentified pleiotropy of our genetic instruments. Genetic instruments for the 25 metabolic traits as an exposure were constructed by developing risk scores using only genome-wide significant SNPs that were not in LD (R^2^ <0.1).[26], [27], [28]

### Phenome-wide association study

We used the SNPs associated with liver iron content and carried out a phenome-wide association study (PheWAS) using publicly available summary statistics from GWASs on predefined ICD10 disease codes, anthropometric traits, and self-reported conditions previously carried out in 452,264 UK Biobank participants of European ancestry,^29^ as well as publicly available, curated summary statistics from previous GWAS (Tables S2–4).^30^ Associations with an FDR <5% were considered statistically significant. A description of how ICD codes were grouped to represent a clinical phenotype are provided elsewhere.^29^

## Results

### The characteristics of liver iron content cohort

The median liver iron content in the UK Biobank was 1.28 mg/g (interquartile range [IQR] 1.16–1.44 mg/g) in men (n = 3,928) and 1.23 mg/g (IQR 1.13–1.38) in women (n = 4,361) (Table 1, Fig. S2). In this cohort, 6.5% of men and 3.4% of women had an elevated liver iron content, above the commonly accepted 1.8 mg/g threshold.^31^ In the IMI DIRECT cohort, the median liver iron content was 1.3 (1.2–1.5) in both men (n = 1,101) and women (n = 412). BMI, waist circumference and diabetes prevalence were lower in the liver iron cohort (n = 8,289) than the remainder of the UK Biobank (n = 402,071) (Table S5). Although invitation was not based on any medical information, MRI exclusion criteria (*e.g.* metal or electrical implants, surgery 6 weeks prior to appointment, severe hearing or breathing problems) may have also contributed to a slightly healthier cohort. The Townsend deprivation index was on average lower in this study cohort. This may be related to MRI participants being biased towards those who live close to the imaging centre (Cheadle) where all of the liver iron cohort were imaged.

**Table 1:** **Characteristics of participants in the UK Biobank and IMI DIRECT study.**

**Characteristics**	**UK Biobank liver iron cohort**		**IMI DIRECT**	
	**Men**	**Women**	**Men**	**Women**
N (%)	3,928	4,361	1,101	412
Age, years (IQR)	57 (51–62)	56 (49–61)	62 (56–66)	62 (57–67)
Liver iron, mg/g (IQR)	1.28 (1.16–1.44)	1.23 (1.13–1.38)	1.3 (1.2–1.5)	1.3 (1.2–1.5)
Waist circumference, cm (IQR)	94 (88–101)	80 (74–89)	101 (95–109)	97 (88–108)
Townsend deprivation index (IQR)	−2.72 (-3.95 to −0.76)	−2.63 (-3.87 to −0.80)	n.a	n.a.
Self-reported diabetes (%)	134 (3.7%)	88 (2.2%)	287 (26%)	216 (52.4%)
BMI, kg/m^2^ (IQR)	26.49 (24.3–29)	25.08 (22.59–28.35)	27.8 (25.8–30.5)	28.7 (25.8–33.2)
No consuming alcohol daily (%)	1,088 (27.7%)	825 (18.9%)	129 (15.6%)	25 (10.7%)

BMI, body mass index; IQR, interquartile range; n.a., not available.

### There are 3 genetic variants associated with liver iron content

**Fig. 2:**
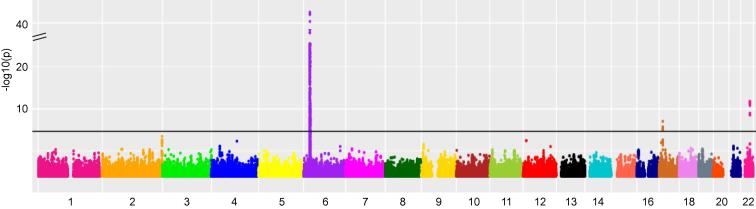
**Manhattan plot illustrating genetic variants associated with liver iron in UK Biobank.** The x-axis is the chromosomal position and y axis is -log(P) for the association with each variant. The black line indicates genome-wide significance level (5 × 10^−8^). SNPs, single-nucleotide polymorphisms.

We performed a GWAS of MRI-derived measures of liver iron content using 8,289 individuals of European ancestry from the UK Biobank (Fig. 2, Fig. S3). We estimated to have more than 80% power in our discovery set to detect variants with MAF ≥5% and effect size ≥0.2 standard deviation (SD) on liver iron content (Fig. S1). We detected no evidence of test-statistic inflation (*λ*_GC_ = 1.016). Our discovery GWAS identified 4 independent variants at *p* <5 × 10^−8^ (Table 2). Two independent variants lie within *HFE*: C282Y (rs1800562; 0.41 SD increase in liver iron per allele; *p* = 5.2 × 10^−42^) and H63D (rs1799945; 0.17 SD; *p* = 8.2 × 10^−15^). The third variant lies in *TMPRSS6*; V736A (rs855791; 0.11 SD; *p* = 1.3 × 10^−11^). The fourth variant, rs149275125, lies between *HS3ST3B1* and *PMP22* (0.41 SD; *p* = 3 × 10^−9^)*.*

**Table 2:** **Genome-wide significant independent variants associated with MRI liver iron content in UK Biobank (*p* <5 × 10^−8^) and validation in IMI DIRECT.**

					**UK Biobank** **(N = 8,289)**				**IMI DIRECT** **(N = 1,513)**				Non-Finnish Europeans*
**SNP**	**Gene**	**Chr**	**EA**	**OA**	**EAF**	**BETA**	**SE**	***p* value**	**EAF**	**BETA**	**SE**	***p* value**	**EAF**
rs1800562	*HFE*	6	A	G	0.08	0.41	0.03	5.2 × 10^−42^	0.04	0.35	0.08	5 × 10^−5^	0.06
rs1799945	*HFE*	6	G	C	0.15	0.16	0.02	8.2 × 10^−15^	0.12	0.19	0.05	2 × 10^−4^	0.14
rs855791	*TMPRSS6*	22	G	A	0.56	0.11	0.02	1.3 × 10^−11^	0.59	0.12	0.04	4 × 10^−4^	0.56
rs149275125	*HS3ST3B1/* *PMP22*	17	C	T	0.98	0.41	0.07	3.1 × 10^−9^	0.99	−0.22	0.2	0.27	0.99

Beta, per allele effect on liver iron (SD); Chr, chromosome; EA, effect allele; EAF, effect allele frequency; OA, other allele; SE, standard error, SNP, single nucleotide polymorphism.

* Data from The Genome Aggregation Database (gnomAD; https://gnomad.broadinstitute.org).

In 1,513 IMI DIRECT participants, we replicated all 3 common variants at *p* <4 × 10^−4^ with a consistent direction of effect and similar effect size (Table S6). The fourth variant, rs149275125, did not associate with liver iron in IMI DIRECT and the direction of effect was opposite to our discovery dataset. This variant is a rare variant (MAF 1%) and has not previously been reported to be associated with any other traits. We focussed all other analyses on the 3 replicated variants.

**Fig. 3:**
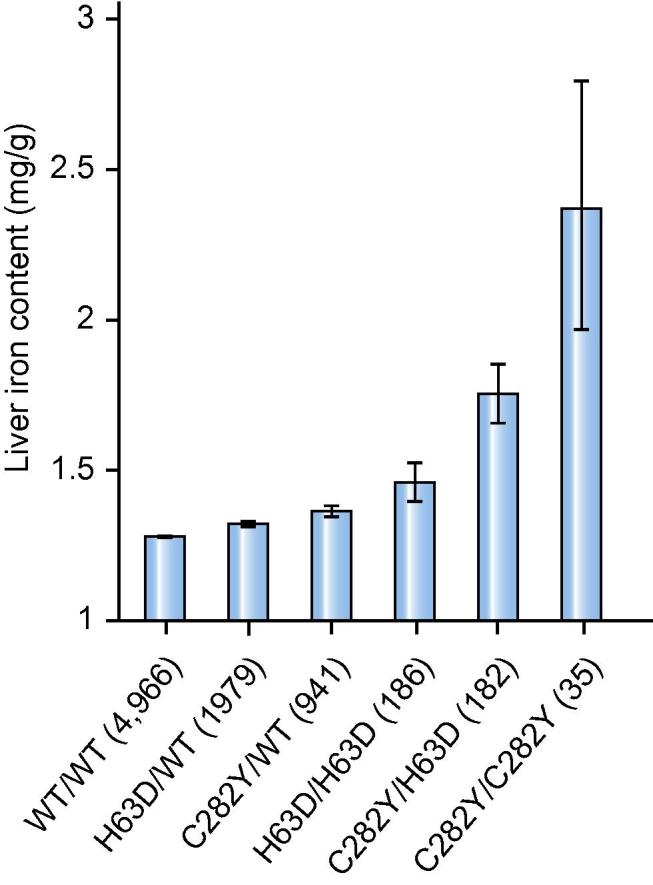
**Liver iron content per genotype group.** The x-axis represents the 6 genotype groups based on the number of C282Y and H63D they carry. The y-axis represents the mean of liver iron (mg/g) per category. Error bars indicate 95% CIs. Numbers in brackets are the number of individuals per genotype category. WT, wild-type.

Both *HFE* and *TMPRSS6* produce proteins that form part of the signalling pathway regulating hepcidin production, the key hormone responsible for iron balance in the body. C282Y homozygotes and C282Y/H63D compound heterozygotes account for ∼85% of cases of hereditary haemochromatosis.^7^ In the UK Biobank, 35 individuals (0.4%) were C282Y homozygotes and had the highest levels of liver iron (mean 2.39 mg/g [±1.2]); 182 (2.2%) were C282Y/H63D compound heterozygotes (1.75 mg/g [±0.7]); 186 (2.2%) were H63D homozygotes (1.46 mg/g [±0.47]); 2,920 (35%) were either C282Y or H63D heterozygotes (1.34 mg/g [±0.32]); 4,966 (60%) did not have any of the variants and had the lowest liver iron (1.28 mg/g [±0.24]) (Fig. 3).

Correlation between the effect sizes and *p* values in the 2 separate GWASs carried out in GEMMA and PLINK showed strong agreement (Figs. S4 and S5). We did not detect any sex-specific variants and the magnitude of effect was similar between men and women (Table S6, Fig. S6). The sensitivity analysis adjusting for alcohol consumption and BMI did not identify any additional signal and did not change the effect size (Table S6). Our pathway analysis demonstrates overlap between liver iron gene sets and pathways involved in autism and schizophrenia (Fig. S7). Nearby genes were visualised with LocusZoom plots (Figs. S8 and S9).

### Liver iron content is heritable and has a high genetic correlation with blood levels of iron biomarkers

We estimated the SNP-based heritability (*h*^2^_SNP_) of liver iron to be 7%. This is similar to heritability estimated for conditions and traits such as coronary artery disease (7%)^32^ and eczema (7%),^33^ but lower than heritability estimated for body fat % (10%)^34^ and transferrin (16%).^11^

To identify genetic overlap between liver iron content and other diseases and traits, we performed LD score regression analyses against a range of available traits and diseases with GWAS summary statistics (448 traits/diseases, Table S7). The most genetically correlated traits were transferrin (r_G_ = −0.78, *p* = 0.04) and ferritin (r_G_ = 1.24, *p* = 0.05) with nominal significant correlation. Joint disorders (r_G_ = −1.17, *p* = 0.50), hypertrophic cardiomyopathy (r_G_ = −1.11, *p* = 0.35), type 2 diabetes (r_G_ = 0.44, *p* = 0.17), chronic kidney disease (r_G_ = 0.57, *p* = 0.48), tinnitus (r_G_ = 0.65, *p* = 0.17), polyuria (r_G_ = 0.75, *p* = 0.36), and gout (r_G_ = 0.90, *p* = 0.19) were highly correlated (r_G_ >0.4) but did not reach a nominal significance threshold (*p* >0.05). Metabolic traits including fasting insulin (r_G_ = 0.17, *p* = 0.53), homeostatic model assessment of insulin resistance (r_G_ = 0.37, *p* = 0.48), fasting glucose (r_G_ = 0.01, *p* = 0.97) and coronary artery disease (r_G_ = −0.01, *p* = 0.97) were not genetically correlated with liver iron content.

### Gene-set enrichment analysis did not identify any enriched tissue or pathways

We used MAGMA implemented as part of the FUMA GWAS platform to assess tissue enrichment of genes at associated loci. We did not find any tissue enrichment, but differentially expressed gene sets were enriched in blood vessels, lung, and adipose tissue, although they did not reach a significant threshold following adjustment for multiple testing (Table S8). None of the pathways reached our FDR significance threshold (Table S9).

### Mendelian randomisation analysis provides evidence for a causal link between central obesity and liver iron content

**Fig. 4:**
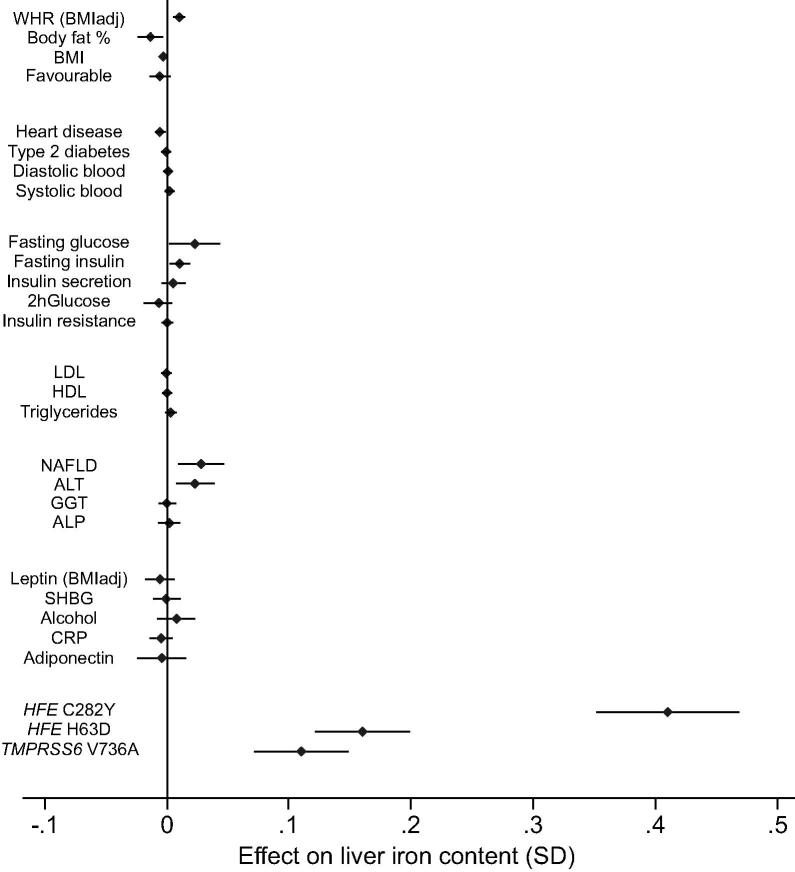
**The effect of 25 predominantly metabolic traits and diseases on liver iron content.** The plot illustrates per allele effect of genetic variants associated with different metabolic traits. We have used alleles associated with higher adiposity, higher metabolic disease risk, higher glycaemic traits, adverse lipid levels, higher liver enzymes and adverse metabolic biomarkers profile. For comparison, the plot illustrates the effect of *HFE* C282Y, *HFE* H63D, *TMPRSS6* V736A on liver iron content. Please refer to Table S10 for the results of 2-sample Mendelian randomisation analysis including 4 circulating iron biomarkers. The error bars indicate 95% CIs. ALP, alkaline phosphatase; ALT, alanine aminotransferase; BMI, body mass index; CRP, C-reactive protein; GGT, gamma-glutamyltransferase; HDL, high-density lipoprotein; LDL, low-density lipoprotein; NAFLD, non-alcoholic fatty liver disease; SHBG, sex hormone binding globulin. The statistical test illustrated on x-vector is from the Inverse Variance Weighted (IVW) method.

We examined the potential causal effect of 25 metabolic traits and diseases (Fig. 4, Table S10) on liver iron content. Following correction for multiple testing (FDR <5%), we found evidence of a causative effect of central obesity, as measured by higher waist-to-hip ratio (adjusted for BMI), on elevated liver iron content (IVW *p* = 0.003) (Table S9, Fig. S10). There was suggestive evidence that higher fasting glucose (IVW *p* = 0.03), higher NAFLD (IVW *p* = 0.04) and higher alanine aminotransferase (IVW *p* = 0.05) were causally associated with higher liver iron content, but none of these associations reached our multiple testing threshold of being statistically significant. All the results were robust to a range of Mendelian randomisation sensitivity analyses.

As a positive control, elevated transferrin saturation levels (IVW *p* = 0.0007), blood iron content (IVW *p* = 0.01) and ferritin levels (IVW *p* = 0.01) were associated with higher liver iron content. The genetic variants (between 5 to 6 variants) associated with these serum iron parameters include the 3 variants associated with liver iron content (*HFE* C282Y, *HFE* H63D, *TMPRSS6* V736A). Therefore, it was not possible to test the causal effect of these parameters on liver iron content independently of *HFE* and *TMPRSS6* genetic variants.

### PheWAS identifies novel associations of liver iron variants with traits and diseases

The 3 liver iron content variants had previously been reported to be associated with multiple haematological parameters, glycated haemoglobin, lipid and bilirubin levels, as well as blood pressure traits (Table S11). We performed a hypothesis-free PheWAS to investigate the association of these 3 variants with other traits and diseases using predefined ICD10 codes, self-reported conditions and traits from the UK Biobank and publicly available GWAS.

**Fig. 5:**
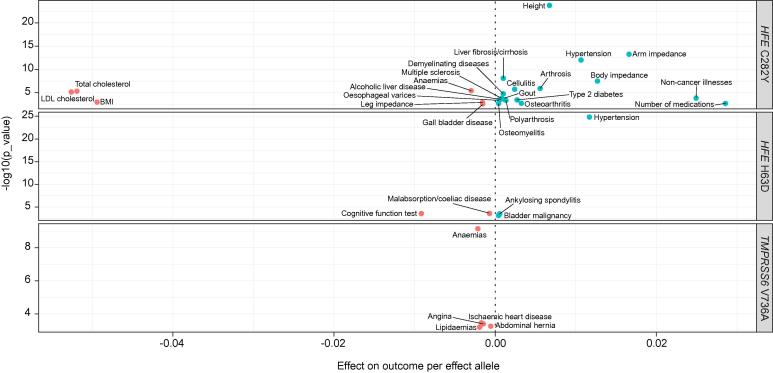
**Illustration of prioritised associations following phenome-wide association studies of rs1800562, rs1799945 and rs855791 and significant traits.** Data from the UK Biobank and publicly available summary statistics. Blue indicates a positive association and red an inverse association, following correction for multiple testing (false discovery rate <5%). Continuous traits betas were scaled to per SD where appropriate for better visualisation. Effect on disease risk is given in log(odds ratio). BMI, body mass index; LDL, low-density lipoprotein.

*HFE* C282Y was associated with higher liver fibrosis/cirrhosis, higher risk of type 2 diabetes, hypertension, alcohol-related liver disease, arthrosis, chronic and degenerative neurological conditions including multiple sclerosis, arthritis and higher height but lower total cholesterol, lower low-density lipoprotein cholesterol (LDL-C) and lower BMI (FDR <5%, Fig. 5, Table S2). *HFE* H63D was associated with higher risk of hypertension, ankylosing spondylitis and bladder malignancy but lower risk of malabsorption or coeliac disease and lower cognitive ability (FDR <5%, Fig. 5^,^ Table S3). The liver iron increasing allele at *TMPRSS6* was associated with lower risk of ischaemic heart disease, angina pectoris, and lipidaemias (FDR <5%, Fig. 5^,^ Table S4).

## Discussion

We performed the first GWAS of multiparametric MRI-determined liver iron content in an unselected population. The identification of loci implicated in increased iron absorption (*HFE* and *TMPRSS6*) provides genetic validation of the utility of MRI for the non-invasive assessment of liver iron content.

The 3 independent variants in *HFE* and *TMPRSS6* have previously been reported to be associated with circulating iron traits including transferrin saturation, blood iron, ferritin, and transferrin levels.^11^ Both *HFE* and *TMPRSS6* play a major role in iron homeostasis by modulating the expression of hepcidin production by the liver.[35], [36] Hepcidin inhibits iron transport and absorption from the gut into the circulation by binding to the main iron transport channel expressed on the surface of duodenal enterocytes, ferroportin.^37^ *TMPRSS6* encodes matriptase 2 (MTP-2), a liver serine protease, which inhibits hepcidin leading to higher iron absorption and bioavailability. *In vitro* studies have shown that major allele at rs888571 inhibits hepcidin more effectively than the missense variant rs888571 (V736A).^38^ *HFE* is a positive upstream regulator of hepcidin transcription. Missense variants C282Y and H63D in *HFE* result in lower hepcidin responsiveness to iron, leading to relative or absolute hepcidin deficiency and subsequent increases in iron absorption and bioavailability.^39^

Elevated liver iron is observationally associated with multiple metabolic traits and diseases in a common condition described as DIOS.^10^ Our Mendelian randomisation analysis supports a causal role for higher central obesity on higher liver iron content, providing further evidence for DIOS. Other traits such as fasting glucose, NAFLD, and alanine aminotransferase showed suggestive causal associations. Animal studies have suggested a putative mechanism of defective iron handling and subsequent iron overload caused by an inflammatory shift and cytokine secretion by activated macrophages that accumulate around adipocytes in obesity.[40], [41] The underlying mechanism, however, is still unclear, and is likely to involve a complex interplay between diet and genetic factors, as well as cross-talk between the liver and visceral adipose tissue.^10^

Our GWAS study identified variants that are likely to regulate iron stores systemically and are not specific to the liver. Therefore, we were not able to examine the causal role of higher liver iron content *per se* on other diseases and traits using Mendelian randomisation. To investigate the phenotypic architecture and shared pathological mechanisms of higher liver iron content with other traits and diseases, we carried out a PheWAS of the 3 genome-wide significant variants against all available disease outcomes and traits from the UK Biobank^29^ and publicly available genetic summary statistics.^30^

Our PheWAS indicates *HFE* C282Y is associated with arthrosis, coxarthrosis, osteoarthritis, and gout, and *HFE* H63D is associated with ankylosing spondylitis and has a suggestive association with dorsalgia. The association between *HFE* C282Y and higher risk of cellulitis, abscesses, furuncles and curbuncles, subcutaneous infections as well as osteomyelitis provides further evidence that genetically elevated iron levels are associated with higher infection risk. Some infectious disease agents are more virulent in an environment with excess iron. There is also evidence that iron overload compromises the ability of phagocytes to kill microorganisms.^42^

Higher iron is correlated with carcinogenesis.^43^ An important mechanism may be oxidative stress and the catalytic activity of iron in the formation of hydroxyl radicals. Iron may also suppress host defences and promote cancer cell proliferation. A recent study found an association between *HFE* C282Y and a higher risk of breast cancer, colorectal cancer, hepatocellular carcinoma, and total cancer.^44^ We found additional evidence of associations with extrahepatic malignancies including bladder cancer (odds ratio 1.0004 per copy of H63D, *p* = 4.7 × 10^−6^, FDR 0.03) and renal cancer (odds ratio 1.0005 per copy of C282Y, *p* = 0.004, FDR 0.05). Despite very small effects, these findings provide genetic evidence for shared mechanisms underlying higher liver iron and extrahepatic cancers.

The association between *HFE* C282Y and neurological conditions such as multiple sclerosis and epilepsy is consistent with the role of iron in many important processes in the central nervous system, including oxygen transportation, oxidative phosphorylation, myelin production, and the synthesis and metabolism of neurotransmitters. In a recent GWAS of brain MRI scans, *HFE* C282Y was associated with iron accumulation in certain parts of the brain.^45^ Observational studies show that iron accumulation in the brain is associated with multiple sclerosis, parkinsonism and Alzheimer’s disease.[46], [47] Individuals with hereditary haemochromatosis frequently develop psychological symptoms, including extreme fatigue, irritability and depression.^48^ Our pathway analysis demonstrates overlap with gene sets and pathways involved in autism and schizophrenia. *HFE* H63D was associated with a reduction in reaction time in specific cognitive function tests, providing further evidence that iron accumulation may cause premature, and indeed preventable, cognitive decline.

The association between *HFE* variants and hypertension is consistent with previous findings.^49^ Possible mechanisms include increased vascular tone secondary to the generation of reactive oxygen species and oxidative stress,^50^ or excess iron accumulation in renal arterioles leading to activation of the renin-angiotensin aldosterone system. We further validated the known association between *HFE* C282Y and type 2 diabetes which could be, at least partly, due to iron accumulation in the pancreas.

The liver iron increasing allele at *TMPRSS6* rs855791 was associated with lower LDL-C, lower risk of angina and ischaemic heart disease. Similar observations have been reported in *HFE* C282Y homozygotes.^51^ A Mendelian randomisation study has recently reported that elevated circulating iron may have a causal (protective) effect on coronary artery disease.^52^ The underlying mechanism is unclear. It is possible that part of this effect is driven through effects on haematological parameters, or lower LDL-C. In conditions where excess iron stores are treated (*e.g.* hereditary haemochromatosis), further research is needed on whether LDL-C levels subsequently increase, and whether the risk of coronary artery disease can be kept low with statins and other preventive interventions.^51^

This study is limited in that the UK Biobank MRI cohort is not a completely unbiased sample of the population. The UK Biobank MRI cohort is slightly more healthy, wealthy, and well educated than the whole cohort of 40–69 year olds in the UK.^53^ The population studied in this work has a slightly lower average BMI and waist circumference than the UK Biobank population as a whole. Larger GWAS studies (*e.g.* on completion of the full 100,000 UK Biobank imaging cohort) may elucidate further susceptibility loci. Ongoing development and validation of MRI scores that may allow accurate determination of the level of inflammation and fibrosis, may lead to further genetic studies focussing on the more severe spectrum of liver disease.

### Conclusion

We performed a large GWAS of MRI liver iron content and identified 3 susceptibility loci previously linked with circulating iron traits. We provided genetic validation for multiparametric MRI as a novel, non-invasive and radiation free imaging modality for liver iron content. Our genetic study suggests that higher liver iron content may be caused in part by higher central adiposity. The deposition of excess iron in the liver seems to share common mechanisms with circulating iron accumulation, which eventually results in widespread damage to parenchymal tissue, leading to several pathologies through a common mechanism.

## Financial support

H.Y. is funded by a Diabetes UK RD Lawrence fellowship (17/0005594). C.A.P is funded by a Wellcome Trust Clinical PhD Programme (206274/Z/17/Z). H.W is funded by an Innovate UK Knowledge Transfer Partnership (KTP10271). The work undertaken by P.W.F was supported in part by ERC-2015-CoG_NASCENT_681742. IMI DIRECT was supported by the Innovative Medicines Initiative Joint Undertaking under grant agreement n°115317 (DIRECT), resources of which are composed of financial contribution from the European Union's Seventh Framework Programme (FP7/2007–2013) and EFPIA companies’ in-kind contribution. H.H is a National Institute for Health Research (NIHR) Senior Investigator.

## Conflict of interest

M.K and R.B. are employees and shareholders of Perspectum Diagnostics. H.W. and S.N. are shareholders in Perspectum Diagnostics. The IMI DIRECT study was funded in part by Boehringer Ingelheim, Eli Lilly, Novo Nordisk, Sanofi Aventis, and Servier. No other potential conflicts of interest relevant to this article were reported.

Please refer to the accompanying ICMJE disclosure forms for further details.

## Authors’ contributions

H.W, C.A.P, H.Y were involved in the study conception and design, analysis and writing of the manuscript. H.H, A.D.H, R.S.P edited the manuscript and supervised C.A.P. E.L.T, J.B, edited the manuscript and wrote elements of the discussion. S.N, R.B, M.K, edited the manuscript and provided the infrastructure underlying the MRI liver iron content measurements. P.W.F, A.M, N.A contributed to the analysis of the validation cohort (IMI DIRECT).

## Ethical approval

This research has been conducted using data from the UK Biobank resource and carried out under UK Biobank project application numbers 9914 and 31037. UK Biobank protocols were approved by the National Research Ethics Service Committee.

## Patient consent

No participants were directly involved in our study, as we used data derived from the UK Biobank study, under project application numbers 9914 and 31037. For the UK Biobank overall study, participants signed written informed consent, specifically applicable to health-related research. All ethical regulations were followed. No patients or participants were specifically or directly involved in setting the research question or the outcome measures or in developing plans for recruitment, design, or implementation of this study. No patients were asked to advise on interpretation or drafting of results. There are no specific plans to disseminate the research results to study participants, but the UK Biobank disseminates key findings from projects on its website.

## Data Availability

Full summary statistics of the MRI liver iron genome-wide association study will be publicly available via UK Biobank.
