# Supplementary material for: Genetic studies of abdominal MRI data identify genes regulating hepcidin as major determinants of liver iron concentration
Source: J Hepatol. 2019 Sep;71(3):594–602. doi: 10.1016/j.jhep.2019.05.032 (PMC6694204; doi:10.1016/j.jhep.2019.05.032)
Supplement: Supplementary Data 1 [file mmc1.pdf]

Genetic studies of abdominal MRI data identify genes regulating hepcidin as major determinants of liver iron concentration

Henry R. Wilman, Constantinos A. Parisinos, Naeimeh Atabaki-Pasdar, Matt Kelly, Elizabeth L Thomas, Stefan Neubauer, IMI DIRECT Consortium, Anubha Mahajan, Aroon D. Hingorani, Riyaz S. Patel, Harry Hemingway, Paul W. Franks, Jimmy D. Bell, Rajarshi Banerjee, Hanieh Yaghootkar

Table of contents

Collaborators.....2
Fig. S1.....5
Fig. S2.....6
Fig. S3.....7
Fig. S4.....8
Fig. S5.....9
Fig. S6.....10
Fig. S7.....11
Fig. S8.....12
Fig. S9.....13
Fig. S10.....14
Table S1..... 15
Table S2..... 16
Table S3..... 21
Table S4..... 24
Table S5..... 27
Table S6..... 28
Table S7..... 30
Table S8..... 31

Table S9……………………………………………………………………………………….. 32
Table S10………………………………………………………………………………………33
Table S11………………………………………………………………………………………35

Collaborators/ Investigators: We would like to thank the following collaborators who work with the IMI DIRECT consortium: Christopher Jennison, Beate Ehrhardt, Patrick Baum, Coria Schoelsch, Jan Freijer, Rolf Grempler, Ulrike Graefe-Mody, Anita Henige, Christiane Dings, Thorsten Lehr, Nina Sihinecich, Francois Pattou, Violetta Raverdi, Robert Caiazzo, Fanelly Torres, Helene Verkindt, Andrea Mari, Toni Giorgino, Roberto Bizzotto, Philippe Froguel, Amelie Bonneford, Mickael Canouil, Veronique Dhennin, Caroline Brorsson, Sonek Brunak, Federico De Masi, Valborg Gudmundsdottir, Helle Pedersen, Karina Banasik, Cecilia Thomas, Peter Sackett, Hans-Henrik Staerfeldt, Agnete Lundgaard, Birgitte Nilsson, Agnes Nielsen, Gianluca Mazzoni, Tugce Karaderi, Simon Rasmussen, Joachim Johansen, Rosa Allesoe, Andreas Fritsche, Barbara Thorand, Jurek Adamski, Harald Grallert, Mark Haid, Sapna Sharma, Martinal Troll, Jonathan Adam, Jorge Ferrer, Heather Eriksen, Gary Frost, Ragna Haussler, Mun-gwan Hong, Jochen Schwenk, Mathias Uhlen, Claudia Nicolay, Imre Pavo, Birgit Steckel Hamann, Melissa Thomas, Kofi Adragni, Han Wu, Leen ‘t Hart, Slieker Roderick, Nienke van Leeuwen, Koen Dekkers, Francesca Frau, Johann Gassenhuber, Bernd Jablonka, Petra Musholt, Hartmut Ruetten, Joachim Tillner, Tania Baltauss, Oana Bernard Poenaru, Nathalie de Preville, Marianne Rodriquez, Manimozhiyan Arumugam, Kristine Allin, Line, Engelbrechtsen, Torben Hansen, Tue Hansen, Annemette Forman, Anna Jonsson, Oluf Pedersen, Avirup Dutta, Josef Vogt, Henrik Vestergaard, Markku Laakso, Tarja Kokkola, Teemu Kuulasmaa, Paul Franks, Nick Giordano, Hugo Fitipaldi, Pascal Mutie, Maria Klintenberg, Margit Bergstrom, Leif Groop, Martin Ridderstrale, Naeimeh Atabaki Pasdar, Harshal Deshmukh, Alison Heggie, Dianne Wake, Donna McEvoy, Ian McVittie, Mark Walker, Andrew Hattersley, Anita Hill, Angus Jones, Timothy McDonald, Mandy Perry,

Rachel Nice, Michelle Hudson, Claire Thorne, Emmanouil Dermitzakis, Ana Vinuela, Louise Cabrelli, Heather Loftus, Adem Dawed, Louise Donnelly, Ian Forgie, Ewan Pearson, Colin Palmer, Andrew Brown, Robert Koivula, Agata Wesolowska-Andersen, Moustafa Abdalla, Nicky McRobert, Juan Fernandez, Yunlong Jiao, Neil Robertson, Stephen Gough, Jane Kaye, Miranda Mourby, Anubha Mahajan, Mark McCarthy, Nisha Shah, Harriet Teare, Reinhard Holl, Anitra Koopman, Femke Rutters, Joline Beulens, Lenka Groeneveld, Anitra Koopman, Brandon Whitcher.

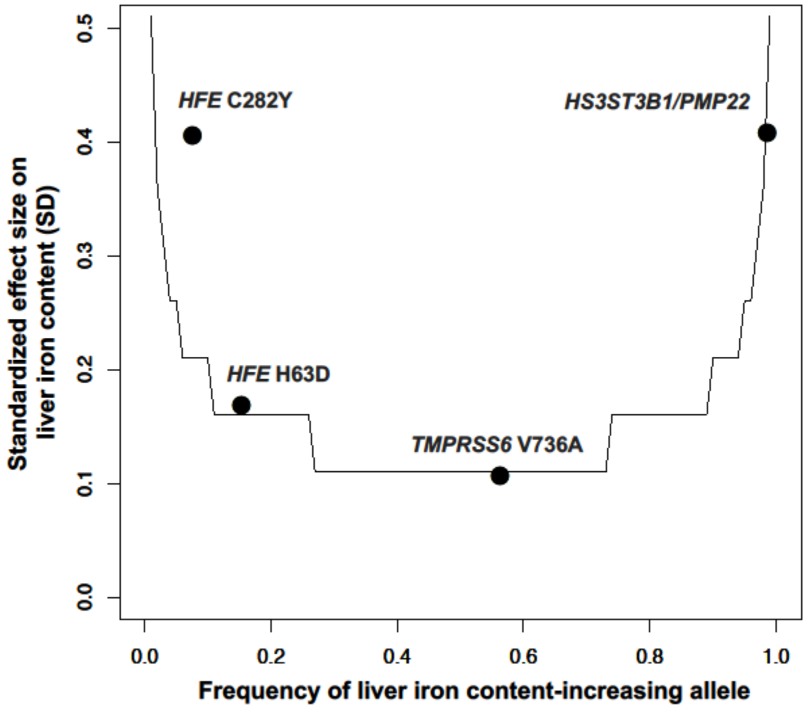

Fig. S1. Power estimates for the GWAS of liver iron content. We used Quanto to calculate our GWAS power in 8,289 individuals of European ancestry from UK Biobank. The line shows 80% power for the minimum standardized effect sizes (in SD units) on liver iron content that could be identified for a given effect-allele frequency at α level 5×10^{-8}. The variants reached GWAS significance level in our discovery set (UK Biobank) are shown in black circles.

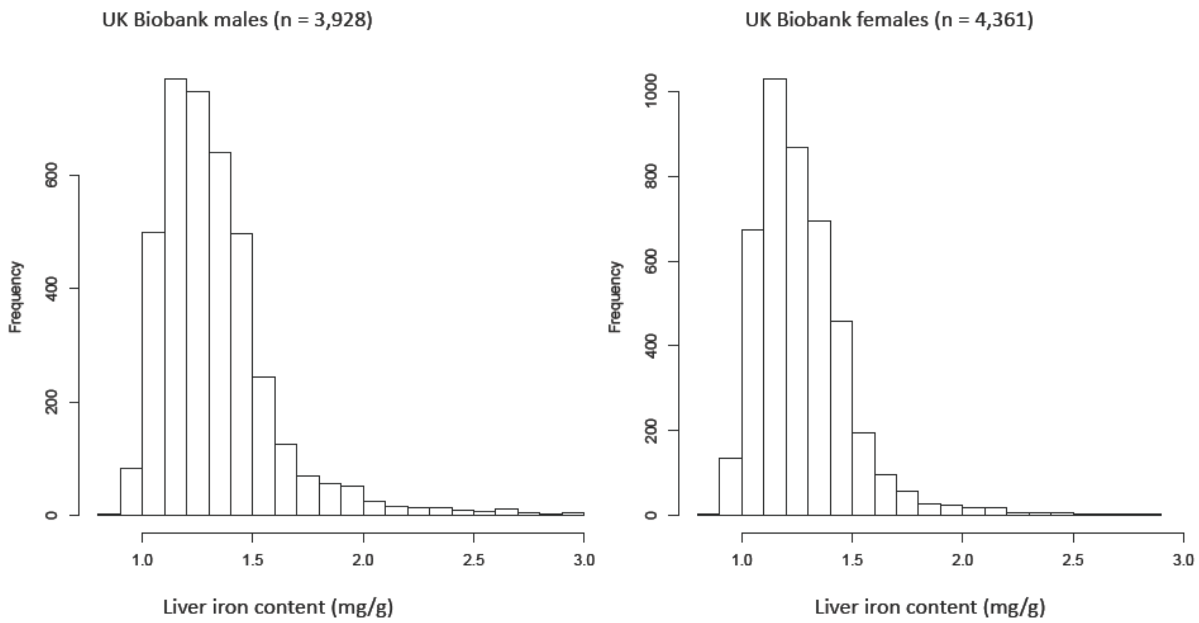

Fig. S2. Histograms of liver iron content distributions in UK Biobank participants, stratified by sex. For better clarity of the histograms, we removed individuals with liver iron content > 3mg/g (33 males and 17 females).

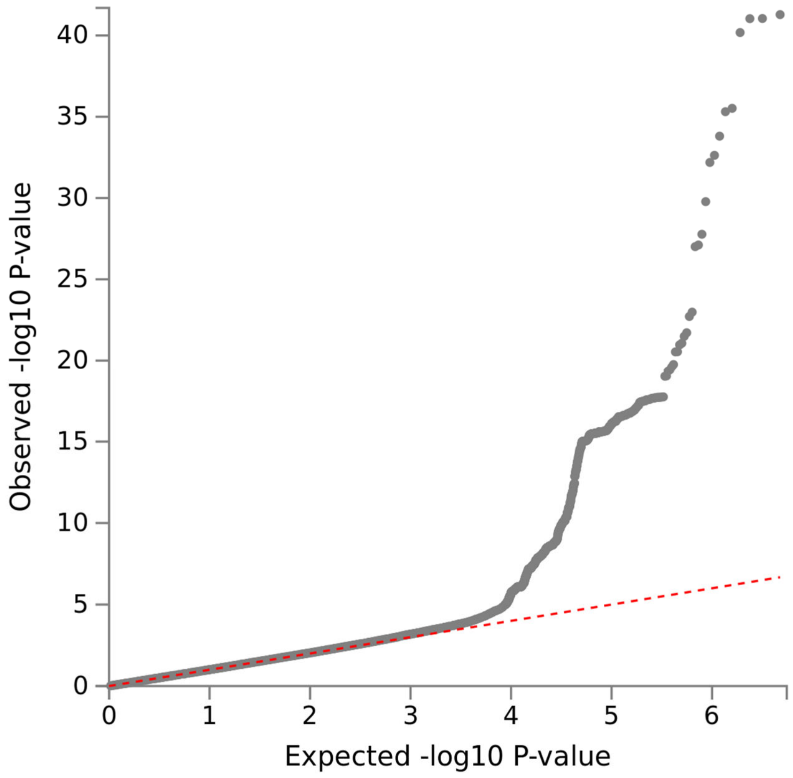

Fig. S3. Quantile quantile (QQ) plot illustrating results of genome wide association study (GWAS) for liver iron in UKB participants (8,289 individuals). This plot llustrates deviation of observed values (black dots) from expected values (null hypothesis, red line).

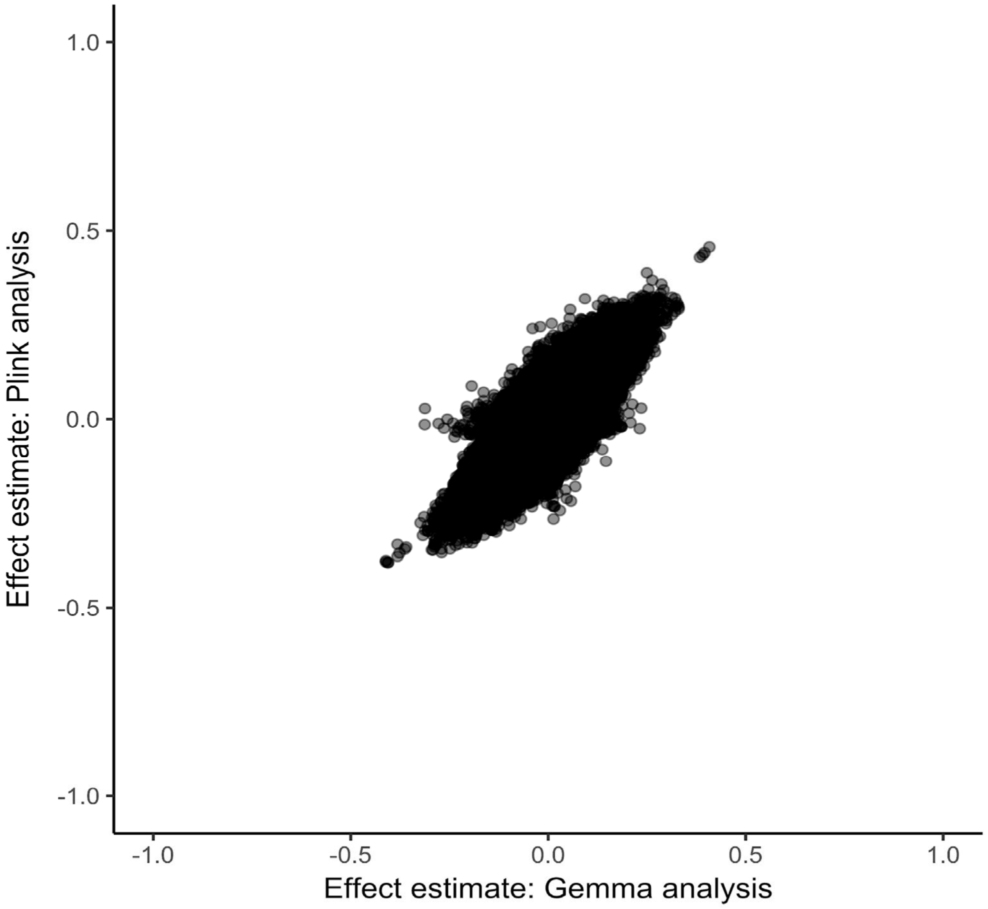

Fig. S4. Correlation plots showing strong agreement between effect estimates from GWAS carried out separately in PLINK and GEMMA. Variants with minor allele frequency < 1 % are not included.

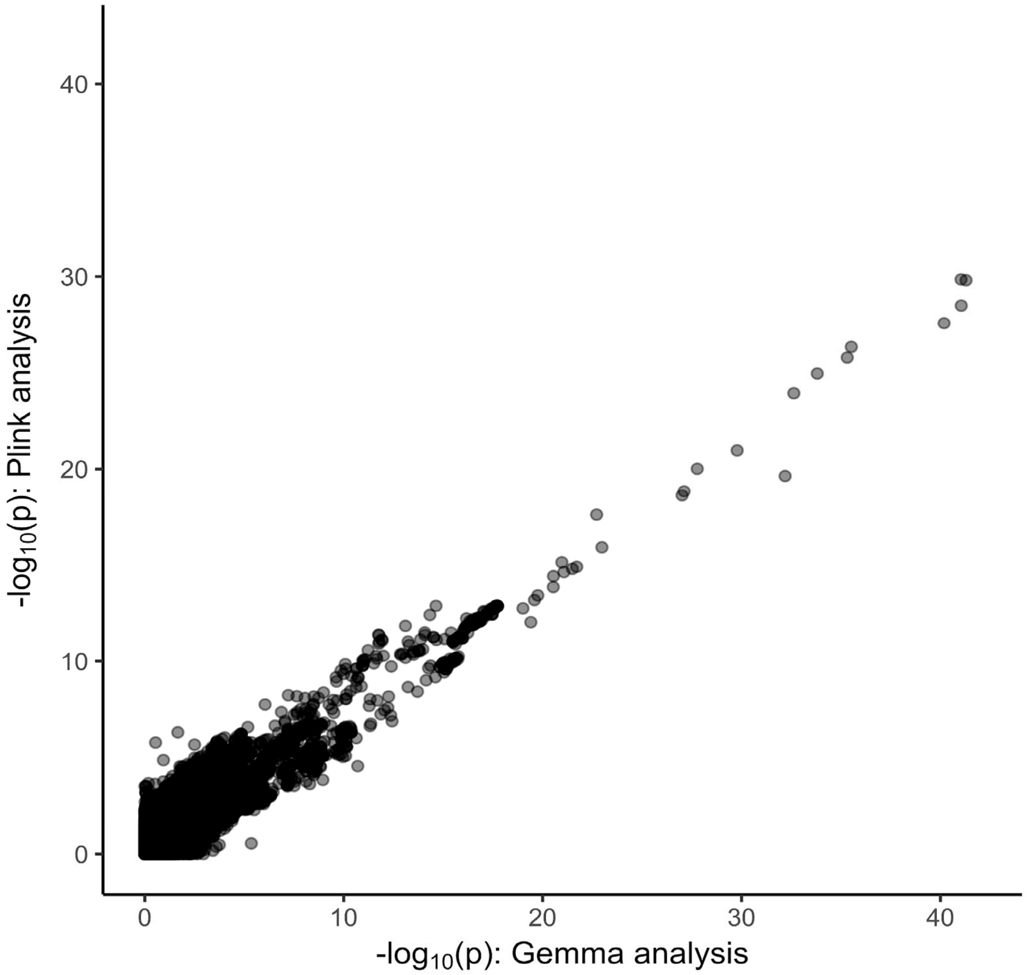

Fig. S5. Correlation plots showing strong agreement between p-value estimates from GWAS carried out separately in PLINK and GEMMA. Variants with minor allele frequency < 1 % are not included.

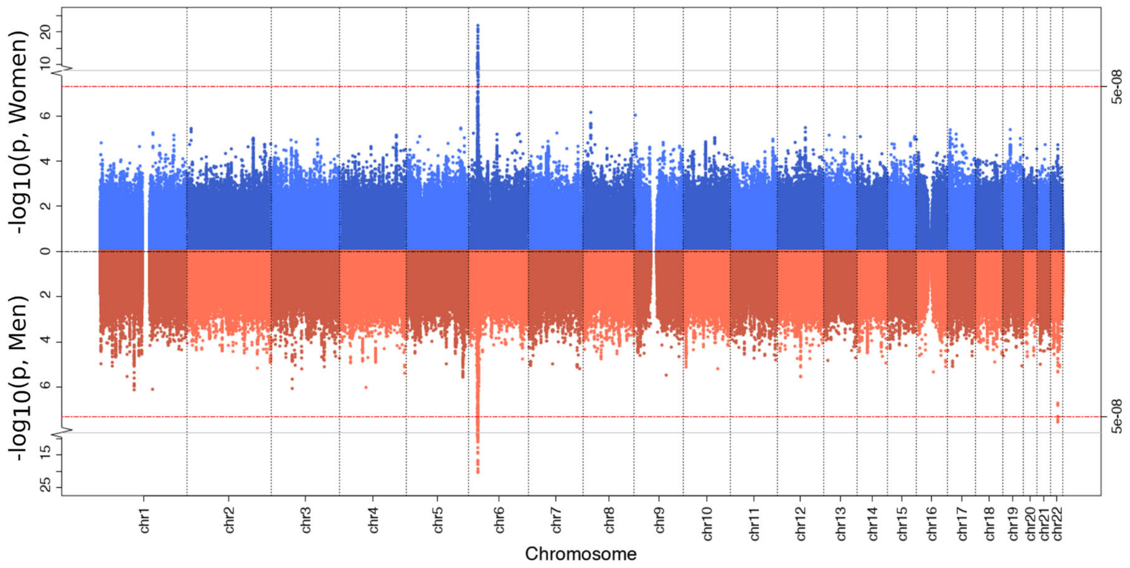

Fig. S6. Gender specific GWAS Manhattan plots do not reveal obvious gender differences.

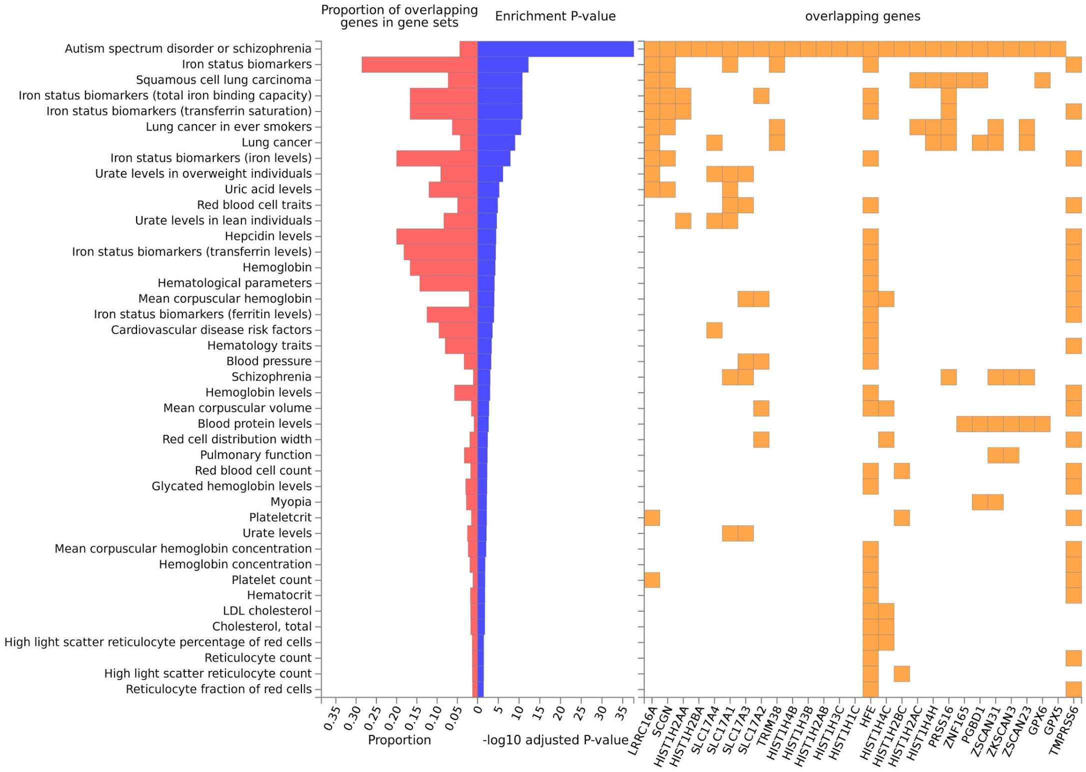

.

Fig. S7. Proportion of overlapping genes in gene sets. High *p*-value enrichment seen with autism spectrum disorder and schizophrenia.

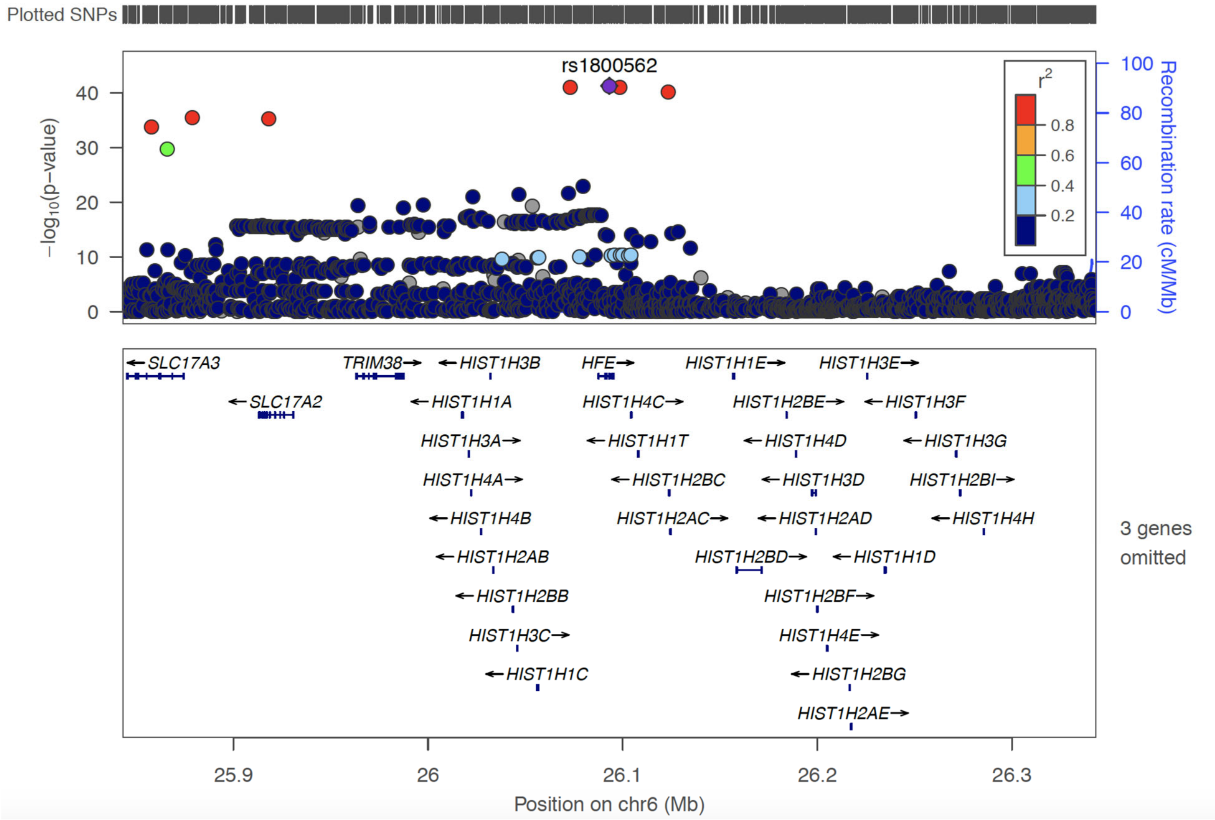

Fig. S8. Locuszoom plot for lead independent locus in *HFE*.

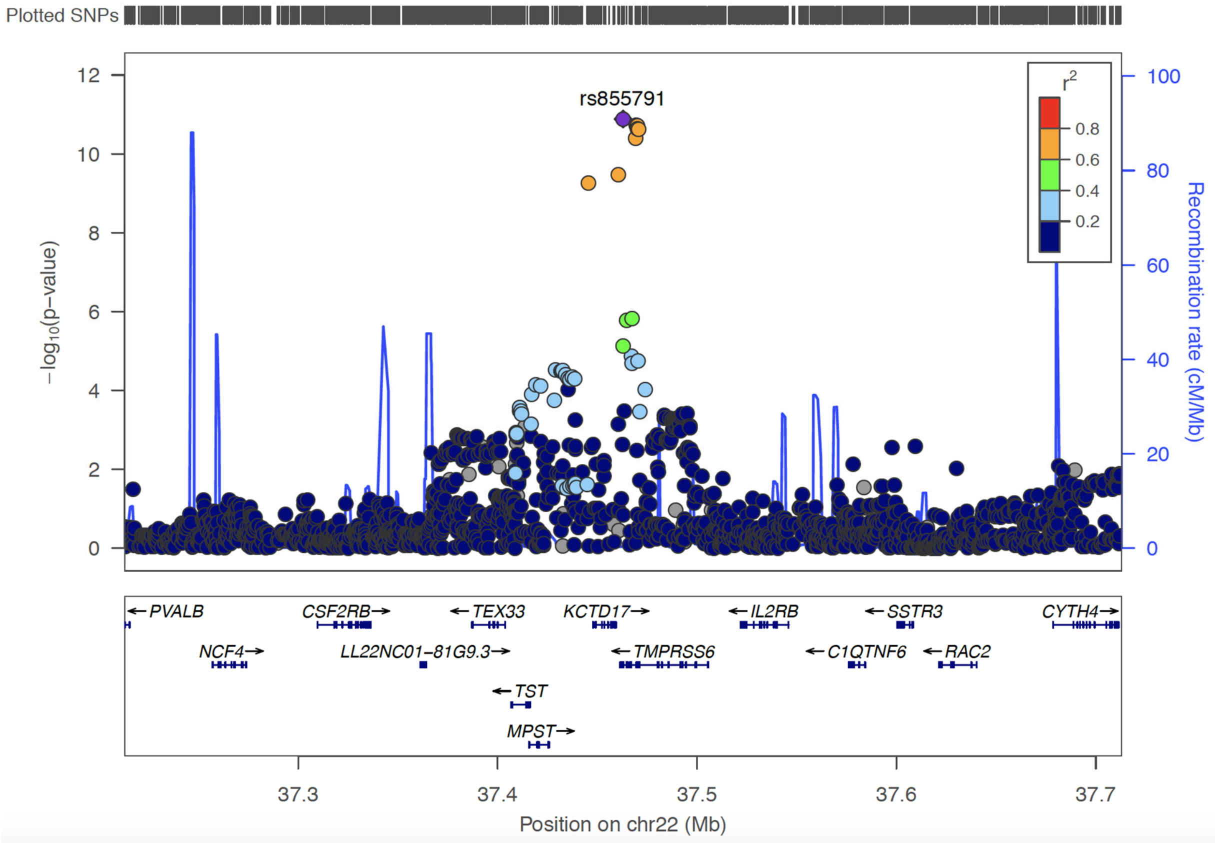

Fig. S9. Locuszoom plot for independent locus in *TMPRSS6*.

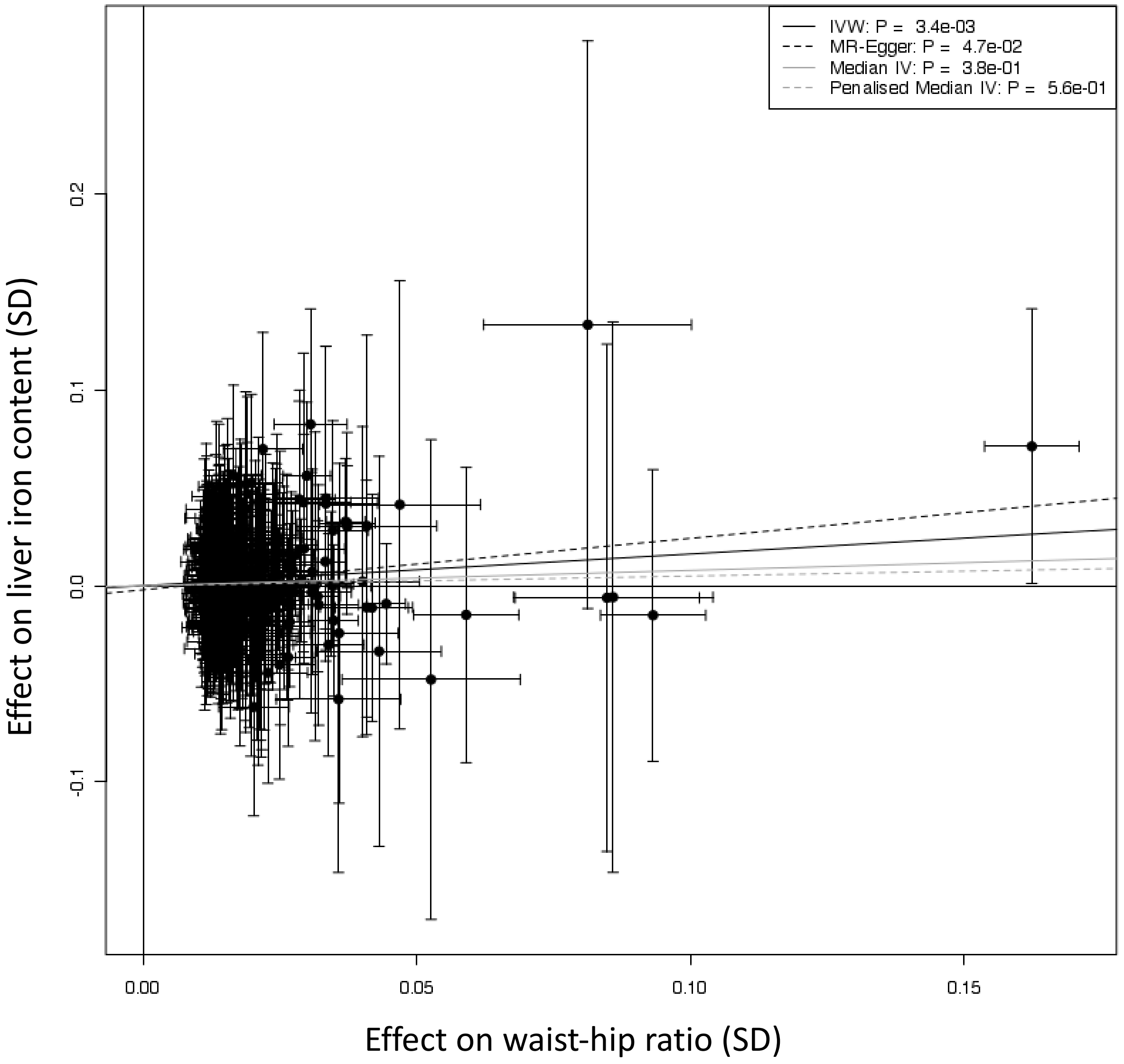

Fig. S10. Mendelian randomisation scatter plot of waist-to-hip ratio adjusted for BMI (WHR BMIadj) vs. liver iron content. Plot shows WHR BMIadj variants and their effects (standard deviation (SD)) on liver iron content (SD). Lines identify the slopes of the four methods tested. Error bars represent standard errors of effect sizes.

Table S1. UK Biobank fields used in main analysis

**UK Biobank Field number**	**Description**
22800-22823	Imputed genotypes (Version 3)
22001	Genetic Sex
22009	Genetic principle components
22000	Genotype measurement batch
21001	BMI (instance 2, at imaging visit)
21003	Age (instance 2, at imaging visit)
31	Sex
22006	Genetic ethnic grouping
22027	Outliers for heterozygosity or missing rate
22021	Genetic kinship to other participants
21000	Ethnic background (self-reported)
22400	Liver Iron
22402	Liver fat percentage

Table S2. Phenome-wide association study (PheWAS) between rs1800562 and 770 traits, disease codes self-reported conditions in UK Biobank and publicly available GWAS summary statistics (we present here, for clarity, traits where $p < 0.05$, Beta = log(odds ratio) per effect allele, FDR = False discovery rate, ICD = Summary statistics from GWAS on UK Biobank ICD codes, SR = Summary statistics derived from GWAS on self reported diseases in UK Biobank, Other_GWAS = Summary statistics outwith UK Biobank, from publicly available summary statistics).

**Trait**	**Beta**	**P_value**	**Category**	**FDR**
Disorders of mineral metabolism	0.013277	< E-200	ICD	1.18E-300
Transferrin Saturation	0.5772	1.52E-178	Other_GWAS	1.18E-175
Transferrin	-0.5496	1.26E-153	Other_GWAS	4.89E-151
Iron	0.3724	3.96E-77	Other_GWAS	1.02E-74
Mean cell haemoglobin	0.4252	6.50E-56	Other_GWAS	1.26E-53
Mean cell volume	0.9425	1.25E-42	Other_GWAS	1.94E-40
Ferritin	0.211	1.43E-29	Other_GWAS	1.85E-27
Standing Height	0.16057	1.89E-24	Traits	2.10E-22
HbA1C	-0.0636	2.59E-20	Other_GWAS	2.51E-18
Comparative height size at age 10	0.019167	2.18E-19	Traits	1.88E-17
Metabolic disorders	0.0093352	2.48E-16	ICD	1.92E-14
Impedance of left arm	0.91402	5.58E-14	Traits	3.94E-12
Haemoglobin concentration	0.1098	5.57E-13	Other_GWAS	3.60E-11
Hypertension	0.010657	9.65E-13	SR	5.76E-11
Impedance of right arm	0.82811	1.48E-12	Traits	8.20E-11
Mean cell haemoglobin concentration	0.0197	2.03E-10	Other_GWAS	1.05E-08
Fibrosis/cirrhosis of liver	0.0010061	7.88E-09	ICD	3.82E-07
Impedance of whole body	1.1305	3.17E-08	Traits	1.45E-06
Comparative body size at age 10	-0.012726	4.21E-08	Traits	1.81E-06
Essential (primary) hypertension	0.0068553	4.99E-07	ICD	2.04E-05
Hypertensive diseases	0.0067368	8.04E-07	ICD	3.08E-05
Coxarthrosis	0.0031267	8.34E-07	ICD	3.08E-05
Other diseases of blood	7.45E-04	8.79E-07	ICD	3.10E-05

Arthrosis	0.005578	1.23E-06	ICD	4.15E-05
Cellulitis	0.0024228	1.78E-06	ICD	5.76E-05
Infections of the skin and subcutaneous tissue	0.0029053	2.18E-06	ICD	6.77E-05
Diseases of veins	0.0052224	2.88E-06	ICD	8.60E-05
Sitting height	0.049223	3.34E-06	Traits	9.60E-05
Other anaemias	-0.0029734	3.63E-06	ICD	1.01E-04
Diseases of liver	0.0019732	3.79E-06	ICD	1.01E-04
Aplastic and other anaemias	-0.0030663	4.12E-06	ICD	1.07E-04
Total cholesterol	-0.0519	4.66E-06	Other_GWAS	1.17E-04
Other diseases of liver	0.0016064	5.56E-06	ICD	1.35E-04
LDL cholesterol	-0.0526	6.91E-06	Other_GWAS	1.62E-04
Demyelinating diseases of the central nervous system	0.001006	1.70E-05	ICD	3.88E-04
Varicose veins of lower extremities	0.0026244	1.79E-05	ICD	3.97E-04
Other arthrosis	0.0028573	1.44E-04	ICD	0.00301969
Number of self-reported non-cancer illnesses	0.024934	1.42E-04	SR	0.00301969
Multiple sclerosis	8.19E-04	1.65E-04	ICD	0.00336008
Alcoholic liver disease	6.40E-04	2.75E-04	ICD	0.00546821
Gout	0.0012223	3.24E-04	ICD	0.00629452
Non-insulin-dependent diabetes mellitus	0.0027233	3.43E-04	ICD	0.00634325
Oesophageal varices	4.94E-04	3.43E-04	ICD	0.00634325
Polyarthrosis	0.0013382	4.21E-04	ICD	0.00760101
Chronic/degenerative neurological problem	0.0010099	9.15E-04	SR	0.01614539
BMI	-0.049401	0.0010255	Traits	0.01768418
Impedance of right leg	-0.056706	0.0012024	Traits	0.01985239
Impedance of left leg	-0.052419	0.0016392	Traits	0.0265004
Osteoarthritis	0.0032516	0.0017763	SR	0.02813079
Osteomyelitis	4.31E-04	0.0019486	ICD	0.03011489
Number of treatments/medications taken	0.028575	0.0019792	SR	0.03011489
haematology	0.0014072	0.0021074	SR	0.03144889
Gall bladder disease	-0.0015547	0.0023865	SR	0.03494196
Cutaneous abscess, furuncle and carbuncle	8.82E-04	0.0025553	ICD	0.03672061
osteomyelitis	4.56E-04	0.0030174	SR	0.04257277

Maternal care for other conditions predominantly related to pregnancy	0.0013672	0.0032596	ICD	0.04516874
Hand grip strength (right)	-0.07552	0.0037987	Traits	0.05171564
Epilepsy	0.0010596	0.0038821	ICD	0.05193982
Malignant neoplasm of kidney, except renal pelvis	5.02E-04	0.0041326	ICD	0.05271919
Joint disorder	0.0035837	0.0041987	SR	0.05271919
Other dermatitis	6.76E-04	0.0042121	ICD	0.05271919
Scoliosis	5.63E-04	0.004075	ICD	0.05271919
Iron deficiency anaemia	-0.0014948	0.0045075	ICD	0.05552095
Diabetes mellitus	0.0022228	0.004908	ICD	0.0595095
Seborrhoeic keratosis	9.18E-04	0.0062934	ICD	0.07513351
Disorders of vitreous body and globe	-6.82E-04	0.0064438	ICD	0.07576347
Other diseases of gallbladder	-6.93E-04	0.0068246	ICD	0.07904313
Hernia	0.0032157	0.0070088	ICD	0.07998278
Pneumothorax	-4.70E-04	0.0074926	ICD	0.0842646
Other diseases of digestive system	0.0015279	0.0081383	ICD	0.09021887
Nutritional anaemias	-0.0014561	0.0087115	ICD	0.09389061
Spontaneous rupture of synovium and tendon	3.67E-04	0.0085996	ICD	0.09389061
obstetric problem	-9.79E-04	0.0092214	SR	0.09802475
Unspecified maternal hypertension	-7.03E-04	0.0096896	ICD	0.10160986
Coagulation defects, purpura and other haemorrhagic conditions	7.96E-04	0.010151	ICD	0.10364705
Ease of skin tanning	-0.0082678	0.010109	SR	0.10364705
deep venous thrombosis (dvt)	0.0013793	0.010681	SR	0.10764229
Pleural plaque	-4.25E-04	0.01084	ICD	0.1078441
Inguinal hernia	0.0018168	0.011232	ICD	0.11032952
Ulcerative colitis	8.56E-04	0.011667	ICD	0.11259664
Other disorders of bone density and structure	5.03E-04	0.011753	ICD	0.11259664
Other soft tissue disorders, not elsewhere classified	0.0016468	0.01249	ICD	0.11819805
Acquired deformities of fingers and toes	0.0014249	0.014362	ICD	0.13400781
Spondylosis	0.0012694	0.014506	ICD	0.13400781
Phlebitis and thrombophlebitis	9.02E-04	0.01508	ICD	0.13767153
eye trauma	-3.53E-04	0.015467	SR	0.1395627

Oesophagitis	0.001381	0.015892	ICD	0.14174933
Atrophic disorders of skin	-7.70E-04	0.016671	ICD	0.14700791
Hand grip strength (left)	-0.061979	0.017494	Traits	0.15083716
Hayfever/allergic rhinitis	0.0020856	0.017318	SR	0.15083716
Other diseases of urinary system	0.0022962	0.0204	ICD	0.17396044
Other diseases of anus and rectum	0.0020638	0.021017	ICD	0.17727383
Diabetes	0.0017854	0.022166	SR	0.18198008
Glaucoma	8.85E-04	0.022506	SR	0.18198008
Venous thromboembolic disease	0.0013882	0.022513	SR	0.18198008
Prolonged pregnancy	8.55E-04	0.025276	ICD	0.202208
Disorders of vitreous body	-4.93E-04	0.026942	ICD	0.21333665
Chronic nephritic syndrome	3.92E-04	0.027676	ICD	0.21693511
Gout	0.15	0.03010851	Other_GWAS	0.23057851
Hip circumference	0.018	0.03	Other_GWAS	0.23057851
Other disorders of urinary system	0.0017628	0.030308	ICD	0.23057851
Other arthritis	0.0013456	0.034365	ICD	0.25890524
Purpura and other haemorrhagic conditions	5.17E-04	0.036492	ICD	0.26465226
fracture head & neck	4.28E-04	0.036387	SR	0.26465226
Other retinal disorders	-7.46E-04	0.035812	ICD	0.26465226
Spondylopathies	0.0013426	0.036003	ICD	0.26465226
Other disorders of ear, not elsewhere classified	3.18E-04	0.037073	ICD	0.26637637
Infectious arthropathies	2.76E-04	0.038766	ICD	0.27598547
Neoplasms of uncertain or unknown behaviour	5.29E-04	0.039637	ICD	0.27962102
Other disorders of external ear	3.89E-04	0.040379	ICD	0.28119607
Fissure and fistula of anal and rectal regions	-6.95E-04	0.040585	ICD	0.28119607
high cholesterol	-0.0023749	0.044088	SR	0.29241272
kidney stone/ureter stone/bladder stone	6.89E-04	0.043154	SR	0.29241272
Cervical disk disorders	4.74E-04	0.043681	ICD	0.29241272
Trunk fat mass	0.032755	0.043875	Traits	0.29241272
Urinary albumin-to-creatinine ratio	-0.16	0.043	Other_GWAS	0.29241272
Postprocedural endocrine and metabolic disorders, not elsewhere classified	4.38E-04	0.046817	ICD	0.30529405

Disorders of skin appendages	0.0012031	0.046798	ICD	0.30529405
Corrected insulin response	0.1	0.04764	Other_GWAS	0.308072
Haemorrhoids	0.0017526	0.049741	ICD	0.31900013

Table S3. Phenome-wide association study (PheWAS) between rs1799985 and 770 traits, disease codes self-reported conditions in UK Biobank and publicly available GWAS summary statistics (we present here, for clarity, traits where $p < 0.05$, Beta = log(odds ratio) per effect allele, FDR = False discovery rate, ICD = Summary statistics from GWAS on UK Biobank ICD codes, SR = Summary statistics derived from GWAS on self reported diseases in UK Biobank, Other_GWAS = Summary statistics outwith UK Biobank, from publicly available summary statistics).

**Trait**	**Beta**	**P_value**	**Category**	**FDR**
Transferrin Saturation	0.228	2.98E-60	Other_GWAS	2.32E-57
Mean cell haemoglobin	0.2172	4.01E-47	Other_GWAS	1.56E-44
Iron	0.1896	1.65E-42	Other_GWAS	4.27E-40
Mean cell volume	0.4633	2.35E-33	Other_GWAS	4.56E-31
Haemoglobin concentration	0.0938	3.60E-26	Other_GWAS	5.59E-24
Hypertension	0.011698	1.48E-25	SR	1.92E-23
Transferrin	-0.1188	5.59E-17	Other_GWAS	6.20E-15
Mean cell haemoglobin concentration	0.0129	1.50E-12	Other_GWAS	1.46E-10
Essential (primary) hypertension	0.0066033	1.05E-10	ICD	9.06E-09
Hypertensive diseases	0.0065562	1.49E-10	ICD	1.16E-08
Ferritin	0.0589	7.38E-06	Other_GWAS	5.21E-04
HbA1C	-0.0181	1.43E-04	Other_GWAS	0.00925925
Malabsorption/coeliac disease	-7.01E-04	2.47E-04	SR	0.01463202
Meantime to correctly identify matches	-1.0768	2.64E-04	SR	0.01463202
Ankylosing spondylitis	5.58E-04	3.05E-04	SR	0.01578398
Variation in diet	0.0058715	4.21E-04	SR	0.02045647
Malignant neoplasm of bladder	4.27E-04	7.06E-04	ICD	0.03228298
Intestinal malabsorption	-6.85E-04	9.68E-04	ICD	0.04180087
gynaecological disorder (not cancer)	0.0035203	0.0011799	SR	0.0482517
uterine problem	0.0028743	0.0014695	SR	0.05709008
Nutritional anaemias	-0.0012992	0.0017931	ICD	0.0663447
Iron deficiency anaemia	-0.0012222	0.0019474	ICD	0.06877863
Other anaemias	-0.0014475	0.0026304	ICD	0.08886177

Dorsalgia	-0.0015782	0.0028488	ICD	0.0922299
soft tissue inflammation	-3.48E-04	0.003833	SR	0.11746746
urinary frequency / incontinence	4.98E-04	0.0039307	SR	0.11746746
Hand grip strength (right)	0.056029	0.0041738	Traits	0.12011269
Unspecified maternal hypertension	5.74E-04	0.0048111	ICD	0.13350802
AUCins/AUCglu	-0.089	0.007847	Other_GWAS	0.19157421
Aplastic and other anaemias	-0.0013261	0.0078898	ICD	0.19157421
Schizophrenia, schizotypal and delusional disorders	-3.78E-04	0.0077531	ICD	0.19157421
Number of self-reported non-cancer illnesses	0.013108	0.0076262	SR	0.19157421
Viral agents as the cause of diseases classified to other chapters	-2.69E-04	0.011049	ICD	0.25036667
Comparative height size at age 10	0.0040296	0.011564	Traits	0.25036667
Ischemic stroke	0.0589	0.0116	Other_GWAS	0.25036667
other abdominal problem	-8.33E-04	0.011555	SR	0.25036667
Diseases of veins, lymphatic vessels and lymph nodes, not elsewhere classified	0.0021004	0.012054	ICD	0.253134
Other dorsopathies	-0.0015424	0.012395	ICD	0.25344513
Postprocedural endocrine and metabolic disorders, not elsewhere classified	4.10E-04	0.013031	ICD	0.25404015
Other specific joint derangements	-5.74E-04	0.013078	ICD	0.25404015
Inflammatory bowel disease	-0.058599	0.0136	Other_GWAS	0.25773659
Varicose veins of lower extremities	0.0011122	0.01531	ICD	0.25860587
LDL cholesterol	-0.0203	0.01425	Other_GWAS	0.25860587
mumps / epidemic parotitis	3.29E-04	0.015122	SR	0.25860587
Other diseases of urinary system	-0.0018048	0.015036	ICD	0.25860587
Standing height	0.028643	0.015167	Traits	0.25860587
Malignant neoplasm of colon	-5.29E-04	0.01615	ICD	0.26699043
Maternal care for other known or suspected foetal problems	8.52E-04	0.016562	ICD	0.26809738
Insulin at 30 minutes	-0.077	0.017936	Other_GWAS	0.27872544
Urate	0.03714019	0.01769045	Other_GWAS	0.27872544
Chronic ischaemic heart disease	-0.0015585	0.018906	ICD	0.28803847
cerebrovascular disease	8.87E-04	0.023869	SR	0.33905455

Crohn's disease	-0.0729995	0.02376	Other_GWAS	0.33905455
Phobic anxiety disorders	-2.43E-04	0.023984	ICD	0.33905455
Obesity class 1	0.041	0.024	Other_GWAS	0.33905455
menorrhagia (unknown cause)	6.20E-04	0.024994	SR	0.34679175
pneumothorax	3.44E-04	0.025717	SR	0.35056332
AUCins	-0.073	0.027434	Other_GWAS	0.350686
Malignant neoplasms of digestive organs	-7.37E-04	0.028434	ICD	0.350686
colitis/not crohns or ulcerative colitis	-3.29E-04	0.02739	SR	0.350686
Height	0.04034753	0.02761489	Other_GWAS	0.350686
Other diseases of stomach and duodenum	-8.40E-04	0.028112	ICD	0.350686
Neuromuscular dysfunction of bladder, not elsewhere classified	-4.00E-04	0.028245	ICD	0.350686
Number of treatments/medications taken	0.015113	0.029067	SR	0.35289155
Diseases of arteries, arterioles and capillaries	-8.32E-04	0.031678	ICD	0.37293645
Unspecified lump in breast	-3.84E-04	0.031667	ICD	0.37293645
Malignant neoplasm of breast	0.001655	0.034513	ICD	0.3876564
Malignant neoplasm of breast	0.001655	0.034513	ICD	0.3876564
Osteonecrosis	2.26E-04	0.034924	ICD	0.3876564
Acute tonsillitis	2.26E-04	0.03665	ICD	0.39103323
Other disorders of bladder	-8.72E-04	0.036738	ICD	0.39103323
Total cholesterol	-0.0163	0.03608	Other_GWAS	0.39103323
Trunk fat mass	0.025299	0.03785	Traits	0.397425
Trunk fat percentage	0.035428	0.040957	Traits	0.42431452
Gout	0.094	0.04160193	Other_GWAS	0.42532503
Urticaria and erythema	2.98E-04	0.042818	ICD	0.42653315
miscarriage	4.91E-04	0.042474	SR	0.42653315
Malignant neoplasms of urinary tract	3.73E-04	0.045212	ICD	0.43912155
Fissure and fistula of anal and rectal regions	-5.10E-04	0.045165	ICD	0.43912155
Impedance of arm (right)	0.17281	0.048858	Traits	0.46867489
Recurrent and persistent haematuria	2.37E-04	0.049943	ICD	0.47324038

Table S4. Phenome-wide association study (PheWAS) between rs855791 and 770 traits, disease codes self-reported conditions in UK Biobank and publicly available GWAS summary statistics (we present here, for clarity, traits where *p* < 0.05, Beta = log(odds ratio) per effect allele, FDR = False discovery rate, ICD = Summary statistics from GWAS on UK Biobank ICD codes, SR = Summary statistics derived from GWAS on self reported diseases in UK Biobank, Other_GWAS = Summary statistics outwith UK Biobank, from publicly available summary statistics).

**Trait**	**Beta**	**P_value**	**Category**	**FDR**
Transferrin Saturation	0.1921	3.50E-80	Other_GWAS	2.72E-77
Iron	0.1868	4.31E-77	Other_GWAS	1.67E-74
Mean cell haemoglobin	0.1928	1.01E-69	Other_GWAS	2.62E-67
Mean cell volume	0.4257	2.40E-54	Other_GWAS	4.66E-52
Haemoglobin concentration	0.0791	4.65E-40	Other_GWAS	7.23E-38
Mean cell haemoglobin concentration	0.0124	3.11E-17	Other_GWAS	4.03E-15
HbA1C	-0.0271	2.74E-14	Other_GWAS	3.04E-12
Aplastic and other anaemias	-0.0023713	4.96E-11	ICD	4.82E-09
Other anaemias	-0.0021435	7.13E-10	ICD	6.16E-08
Ferritin	0.0513	5.81E-08	Other_GWAS	4.51E-06
Disorders of mineral metabolism	5.92E-04	8.42E-05	ICD	0.00594758
Transferrin	-0.0395	1.29E-04	Other_GWAS	0.00835275
Chronic ischaemic heart disease	-0.0017122	3.48E-04	ICD	0.02077519
Angina pectoris	-0.0014759	4.03E-04	ICD	0.02237483
abdominal hernia	-5.49E-04	5.57E-04	SR	0.02884587
Nutritional anaemias	-0.0010314	6.07E-04	ICD	0.02904152
Lipidaemias	-0.0019186	6.35E-04	ICD	0.02904152
Iron deficiency anaemia	-8.71E-04	0.0022528	ICD	0.09724587
Ischaemic heart diseases	-0.0015564	0.0027755	ICD	0.11350334
Gingivitis and periodontal diseases	-2.70E-04	0.0043173	ICD	0.16772711
Other disorders of brain	-3.50E-04	0.0072543	ICD	0.2684091
Malignant neoplasms of digestive organs	6.40E-04	0.0086194	ICD	0.30442154
chronic/degenerative neurological problem	-4.20E-04	0.010599	SR	0.34751325

Premature rupture of membranes	-5.01E-04	0.010734	ICD	0.34751325
peripheral vascular disease	2.91E-04	0.011528	SR	0.35829024
Lung cancer	-0.045758	0.012554	Other_GWAS	0.37517146
Nasal polyp	-4.55E-04	0.013605	ICD	0.39152167
dermatology	-0.0010167	0.015101	SR	0.41905275
Haemorrhoids	-0.0011652	0.016093	ICD	0.4249672
other abdominal problem	-5.73E-04	0.016408	SR	0.4249672
Unspecified renal colic	-3.38E-04	0.017789	ICD	0.44587268
Coffee intake	0.0096721	0.020428	SR	0.4535016
Comparative height size at age 10	0.0026506	0.01988	Traits	0.4535016
Disorders of muscles	-2.57E-04	0.020078	ICD	0.4535016
Diseases of Bartholin's gland	-3.83E-04	0.018862	ICD	0.4535016
Lung adenocarcinoma	-0.0648425	0.021971	Other_GWAS	0.47420742
Extrapyramidal and movement disorders	-3.27E-04	0.024441	ICD	0.513261
Malignant neoplasm of colon	3.55E-04	0.025919	ICD	0.52202446
Other disorders of muscle	-2.19E-04	0.026202	ICD	0.52202446
eczema/dermatitis	-7.10E-04	0.031241	SR	0.53614689
Parkinson's disease	-2.42E-04	0.028552	ICD	0.53614689
Diseases of tongue	2.49E-04	0.030177	ICD	0.53614689
Redundant prepuce, phimosis and paraphimosis	-6.99E-04	0.031741	ICD	0.53614689
Obesity class 2	0.043	0.028	Other_GWAS	0.53614689
oesophageal disorder	-0.0011093	0.030462	SR	0.53614689
Triglycerides	0.0106	0.02945	Other_GWAS	0.53614689
Bacterial diseases	4.62E-04	0.042392	ICD	0.55084205
Hypothyroidism	-7.82E-04	0.034171	ICD	0.55084205
Other disorders of fluid, electrolyte and acid-base balance	4.80E-04	0.043162	ICD	0.55084205
Demyelinating diseases	-2.62E-04	0.037883	ICD	0.55084205
Other disorders of the nervous system	-4.40E-04	0.035772	ICD	0.55084205
heart/cardiac problem	-0.0010641	0.042489	SR	0.55084205
Other forms of heart disease	-0.0010709	0.03837	ICD	0.55084205
Internalizing problems	-0.0668	0.04297	Other_GWAS	0.55084205
irritable bowel syndrome	6.53E-04	0.04067	SR	0.55084205
Other disorders of teeth and supporting structures	-3.57E-04	0.034685	ICD	0.55084205
Femoral hernia	-1.67E-04	0.042801	ICD	0.55084205

Renal failure	6.15E-04	0.03951	ICD	0.55084205
Unspecified lump in breast	2.62E-04	0.043245	ICD	0.55084205
Number of self-reported cancers	0.0013253	0.04072	SR	0.55084205
Maternal care related to the fetus and amniotic cavity and possible delivery problems	-9.57E-04	0.034907	ICD	0.55084205
other fractures	-1.72E-04	0.044586	SR	0.55876326
Essential (primary) hypertension	-0.0014729	0.045465	ICD	0.560735
Hypertensive diseases	-0.0014694	0.046218	ICD	0.56111541
Other peripheral vascular diseases	-3.94E-04	0.047072	ICD	0.56269145
Cervical disk disorders	-2.51E-04	0.049426	ICD	0.57319406
Complications predominantly related to the puerperium	-2.97E-04	0.04875	ICD	0.57319406

Table S5. Baseline characteristics of UK Biobank participants who took part in the imaging study compared to rest of the UK Biobank cohort (IQR = interquartile range).

	UK Biobank liver iron cohort		UK Biobank rest of cohort	
**Characteristics**	**Men**	**Women**	**Men**	**Women**
**No (%)**	3,928	4,361	184637	217434
**age (IQR) (years)**	57 (51,62)	56 (49,61)	59 (51, 64)	58 (51, 63)
**Waist Circumference (IQR) (cm)**	94 (88, 101)	80 (74, 89)	96 (89, 104)	83.0 (75.2, 92.0)
**Townsend deprivation index (IQR)**	-2.72 (-3.95, -0.76)	-2.63 (-3.87,-0.80)	-2.33 (-3.74, 0.16)	-2.34 (-3.73, 0.03)
**Self reported diabetes (%)**	134 (3.7%)	88 (2.2%)	12102 (6.6%)	7482 (3.5%)
**Liver iron (IQR) (mg/g)**	1.28 (1.155,1.437)	1.23 (1.13,1.38)	NA	NA
**BMI (IQR) (kg/m2)**	26.49 (24.3, 29)	25.08 (22.59,28.35)	27.3 (25.0, 30.1)	26.1 (23.5, 29.7)
**No consuming alcohol daily (%)**	1,088 (27.7%)	825 (18.9%)	48497 (26.3%)	36336 (16.7%)

Table S6. Sensitivity analyses for BMI unadjusted, alcohol adjusted and sex specific analyses in UK Biobank and separate results for participants with or without diabetes in IMI DIRECT (Effect Allele = ALLELE1, Other Allele = ALLELE0, Beta = log(odds ratio) per effect allele, SE = standard error) .

**UK Biobank BMI adjusted**								
**SNP**	**chr**	**pos**	**ALLELE1**	**ALLELE0**	**A1_FREQ**	**BETA**	**SE**	**P**
**rs1800562**	6	26093141	A	G	0.076	4.06E-01	2.94E-02	5.21E-42
**rs1799945**	6	26091179	G	C	1.53E-01	1.69E-01	2.16E-02	8.24E-15
**rs855791**	22	37462936	G	A	5.63E-01	1.07E-01	1.57E-02	1.31E-11
**rs149275125**	17	14831420	C	T	9.86E-01	4.08E-01	6.87E-02	3.14E-09
**UK Biobank Men**								
**SNP**	**chr**	**pos**	**ALLELE1**	**ALLELE0**	**A1_FREQ**	**BETA**	**SE**	**P**
**rs1800562**	6	26093141	A	G	0.076	4.04E-01	4.22E-02	4.72E-21
**rs1799945**	6	26091179	G	C	1.53E-01	1.72E-01	3.15E-02	6.18E-08
**rs855791**	22	37462936	G	A	5.63E-01	1.27E-01	2.30E-02	3.97E-08
**rs149275125**	17	14831420	C	T	9.86E-01	4.48E-01	1.01E-01	9.45E-06
**UK Biobank Women**								
**SNP**	**chr**	**pos**	**ALLELE1**	**ALLELE0**	**A1_FREQ**	**BETA**	**SE**	**P**
**rs1800562**	6	26093141	A	G	0.076	4.04E-01	4.09E-02	2.42E-22
**rs1799945**	6	26091179	G	C	1.53E-01	1.71E-01	2.98E-02	1.15E-08
**rs855791**	22	37462936	G	A	5.63E-01	9.11E-02	2.16E-02	2.57E-05
**rs149275125**	17	14831420	C	T	9.86E-01	3.85E-01	9.37E-02	4.16E-05
**UK Biobank BMI unadjusted**								
**SNP**	**chr**	**pos**	**ALLELE1**	**ALLELE0**	**A1_FREQ**	**BETA**	**SE**	**P**
**rs1800562**	6	26093141	A	G	0.076	4.02E-01	2.93E-02	2.27E-41
**rs1799945**	6	26091179	G	C	1.53E-01	1.75E-01	2.16E-02	8.49E-16
**rs855791**	22	37462936	G	A	5.63E-01	1.07E-01	1.57E-02	1.15E-11
**rs149275125**	17	14831420	C	T	9.86E-01	4.03E-01	6.87E-02	4.71E-09
**UK Biobank alcohol adjusted**								
**SNP**	**chr**	**pos**	**ALLELE1**	**ALLELE0**	**A1_FREQ**	**BETA**	**SE**	**P**
**rs1800562**	6	26093141	A	G	0.04997	4.46E-01	3.66E-02	8.80E-33
**rs1799945**	6	26091179	G	C	0.1196	1.87E-01	2.77E-02	1.90E-11
**rs855791**	22	37462936	G	A	0.59145	1.08E-01	1.99E-02	7.06E-08
**rs149275125**	17	14831420	C	T	9.86E-01	5.09E-01	8.62E-02	4.24E-09
**DIRECT Non-Diabetics only (nr = 1010)**								
**SNP**	**chr**	**pos**	**ALLELE1**	**ALLELE0**	**A1_FREQ**	**BETA**	**SE**	**P**
**rs1800562**	6	26093141	A	G	0.04997	0.34217	0.11693	0.0035

**rs1799945**	6	26091179	G	C	0.1196	0.17716	0.07098	0.0127
**rs855791**	22	37462936	G	A	0.59145	0.18474	0.04387	2.77E-05
**rs149275125**	17	14831420	C	T	0.99069	-0.311771	0.3051	0.307
**DIRECT Diabetics only (nr = 503)**								
**SNP**	**chr**	**pos**	**ALLELE1**	**ALLELE0**	**A1_FREQ**	**BETA**	**SE**	**P**
**rs1800562**	6	26093141	A	G	0.04997	0.32545	0.11389	0.00444
**rs1799945**	6	26091179	G	C	0.1196	0.23808	0.08316	0.00437
**rs855791**	22	37462936	G	A	0.59145	0.01412	0.0641	0.826
**rs149275125**	17	14831420	C	T	0.99069	-0.162332	0.26725	0.544

Table S7. Genetic correlations between liver iron content and 448 LD Hub traits phenotypes ordered by *p*-value (we present here, for clarity, traits where $p < 0.2$, SE = standard error). LD Hub is a centralized database of summary-level GWAS results for 448 diseases and traits from different publicly available resources/consortia and a web interface that automates the LD score regression analysis pipeline. LD score regression is a reliable and efficient method of using GWAS summary level genetic data to estimate the genetic correlation between different phenotypes. We estimated the genetic correlation between liver iron content and 448 diseases and traits available in LD Hub, and, for clarity, present only the traits and phenotype with a p-value < 0.2.

**Trait**	**SE**	**P_value**
**Transferrin**	0.3785	0.0392
**Ferritin**	0.6433	0.0544
**Anorexia Nervosa**	0.1545	0.1061
**Non-cancer illness code_ self-reported: malabsorption/coeliac disease**	0.1948	0.1255
**Diagnoses - main ICD10: M10 Gout**	0.6034	0.1326
**Urate**	0.3247	0.1334
**Non-cancer illness code_ self-reported: kidney stone/ureter stone/bladder stone**	0.3441	0.1675
**Non-cancer illness code_ self-reported: gout**	0.3166	0.1735
**Type 2 Diabetes**	0.3258	0.1776
**Tinnitus: Yes_ but not now_ but have in the past**	0.4983	0.193
**Age started oral contraceptive pill**	0.2231	0.1982

Table S8. GTEx tissue enrichment results from FUMA (MAGMA) (Beta = log(odds ratio), SE = standard error, FDR = False discovery rate).

TISSUE	BETA	SE	P_value	FDR
Blood_Vessel	0.024	0.0123	0.025484	0.32273
Adipose_Tissue	0.0252	0.0137	0.032273	0.32273
Lung	0.0229	0.0115	0.022835	0.32273
Muscle	0.0111	0.00711	0.058704	0.34373
Blood	0.0105	0.00669	0.059211	0.34373
Nerve	0.0171	0.0115	0.068746	0.34373
Heart	0.0135	0.0097	0.082246	0.35248286
Breast	0.0175	0.0166	0.1459	0.547125
Fallopian_Tube	0.0145	0.0152	0.17044	0.56813333
Spleen	0.00662	0.00803	0.20486	0.61458
Testis	0.00382	0.00551	0.24447	0.66673636
Liver	0.00217	0.00679	0.37473	0.88763077
Thyroid	0.0034	0.0116	0.38464	0.88763077
Uterus	-0.0033	0.013	0.59997	0.97684
Brain	-0.00261	0.00707	0.64422	0.97684
Cervix_Uteri	-0.00923	0.0167	0.71013	0.97684
Skin	-0.00817	0.0112	0.76778	0.97684
Adrenal_Gland	-0.00832	0.0104	0.78835	0.97684
Ovary	-0.00929	0.0107	0.8071	0.97684
Bladder	-0.0132	0.015	0.81052	0.97684
Esophagus	-0.0166	0.0172	0.8333	0.97684
Vagina	-0.015	0.0135	0.86674	0.97684
Small_Intestine	-0.0109	0.0097	0.86847	0.97684
Salivary_Gland	-0.0144	0.0116	0.89176	0.97684
Colon	-0.0221	0.0167	0.9073	0.97684
Stomach	-0.0194	0.0144	0.91025	0.97684
Prostate	-0.0218	0.0141	0.93928	0.97684
Kidney	-0.0172	0.0106	0.94844	0.97684
Pancreas	-0.0178	0.00954	0.96913	0.97684
Pituitary	-0.0165	0.00829	0.97684	0.97684

Table S9. Gene-set enrichment results from FUMA (MAGMA) on 10,651 gene-sets (for clarity, we are presenting enriched pathways with *p* values < 0.001, Beta = log(odds ratio), SE = standard error, FDR = False discovery rate).

Pathway	Number of Genes	Beta	SE	P_value	FDR
**GO_bp:go_mitochondrial _calcium_ion_transport**	11	0.988	0.259	6.92E-05	0.56
**Curated_gene_sets: chassot_skin_wound**	10	1.05	0.284	1.10E-04	0.56
**Curated_gene_sets: wu_hbx_targets_2_up**	21	0.681	0.192	2.02E-04	0.56
**Curated_gene_sets: yang_muc2_targets _duodenum_6mo_dn**	20	0.581	0.165	2.10E-04	0.56
**GO_bp:go_cellular_hormone _metabolic_process**	99	0.27	0.0792	3.21E-04	0.61
**Curated_gene_sets:kim _germinal_center_t_helper_up**	61	0.37	0.11	3.83E-04	0.61
**GO_bp:go_establishment _of_spindle_orientation**	26	0.498	0.149	4.27E-04	0.61
**GO_bp:go_uv_protection**	12	0.723	0.218	4.60E-04	0.61
**GO_bp:go_urate_ metabolic_process**	12	0.736	0.227	5.91E-04	0.64
**GO_bp:go_negative_ regulation_of_interleukin _2_production**	15	0.709	0.219	6.05E-04	0.64
**GO_bp:go_calcium_ion_import**	62	0.334	0.105	7.61E-04	0.65
**GO_bp:go_metal_ion_transport**	545	0.111	0.0357	8.87E-04	0.65
**GO_mf:go_dna_dependent _atpase_activity**	72	0.268	0.0859	8.97E-04	0.65

Table S10. Mendelian randomisation studies of 29 predominantly metabolic traits and liver iron content (as exposures) and liver iron content (as an outcome). Presented here are results from main analysis (inverse variance weighting (IVW)) and sensitivity analyses (Egger, weighted median (WM) and penalised weighted median(PWM)). BMIadj = BMI adjusted, SHBG = sex hormone binding globulin, N SNP = Number of SNPs.

Exposure	Beta IVW	P IVW	Beta Egger	P Egger	Egger int	Int p	Beta WM	P WM	Beta PWM	P PWM	N SNP	Reference
**transferrin saturation**	0.645	0.0007	0.77	0.003	-0.034	0.171	0.68	1.6E-45	0.691	7.0E-49	5	PMID: 25352340
**WHR BMIadj**	0.162	0.003	0.26	0.047	-0.002	0.401	0.08	3.8E-01	0.049	5.6E-01	336	PMID: 30239722
**Iron**	0.833	0.010	1.33	0.012	-0.091	0.096	0.84	1.7E-18	0.794	6.2E-14	5	PMID: 25352340
**Ferritin**	1.512	0.011	2.11	0.051	-0.055	0.412	1.96	1.3E-21	1.69	2.4E-05	6	PMID: 25352340
**Fasting glucose**	0.216	0.037	0.33	0.068	-0.004	0.429	0.35	8.0E-03	0.35	1.2E-02	32	PMID: 22885924
**NAFLD**	0.205	0.040	0.21	0.185	-0.002	0.790	0.21	8.9E-04	0.21	1.5E-03	4	PMID: 21423719
**Body fat %**	-0.360	0.044	0.43	0.518	-0.029	0.171	-0.42	3.7E-02	-0.42	4.7E-02	10	PMID: 26833246
**Alanine transaminase**	0.008	0.050	0.01	0.298	0.000	0.989	0.01	5.8E-03	0.01	4.8E-03	4	PMID: 22001757
**Transferrin**	-0.360	0.058	-0.27	0.284	-0.024	0.577	-0.17	4.4E-02	-0.28	7.3E-02	9	PMID: 25352340
**Fasting insulin BMIadj**	0.740	0.065	2.89	0.163	-0.035	0.281	0.91	3.6E-02	0.92	3.2E-02	12	PMID: 22885924
**Insulin secretion**	0.253	0.065	0.45	0.061	-0.010	0.167	0.38	1.1E-02	0.38	1.3E-02	11	PMID: 24296717
**BMI**	-0.129	0.075	-0.11	0.466	0.000	0.914	0.01	9.5E-01	0.01	9.5E-01	73	PMID: 25673413
**Coronary artery disease**	-0.052	0.077	0.04	0.524	-0.008	0.079	-0.04	3.7E-01	-0.04	3.9E-01	64	PMID: 26343387
**2hGlu**	-0.109	0.232	-0.43	0.239	0.028	0.352	-0.09	3.7E-01	-0.09	3.8E-01	8	PMID: 22885924
**Favourable adiposity**	-0.277	0.326	0.04	0.960	-0.007	0.684	-0.41	1.6E-01	-0.42	1.8E-01	14	PMID: 30352878
**LDL-C**	-0.069	0.350	-0.13	0.267	0.004	0.503	0.03	6.7E-01	0.03	6.6E-01	76	PMID: 24097068
**Leptin BMIadj**	-0.096	0.380	0.33	0.591	-0.026	0.446	-0.11	4.0E-01	-0.11	3.9E-01	5	PMID: 26833098
**Type 2 diabetes**	-0.021	0.393	-0.05	0.264	0.003	0.428	-0.07	6.3E-02	-0.07	8.9E-02	84	PMID: 29632382

**SHBG**	-0.134	0.397	-0.56	0.093	0.024	0.138	-0.38	1.4E-02	-0.38	1.4E-02	10	PMID: 22829776
**Alcohol**	0.440	0.419	2.40	0.400	-0.044	0.477	0.78	1.1E-01	0.78	1.3E-01	6	PMID: 28937693
**HDL-C**	0.033	0.458	0.07	0.281	-0.002	0.431	0.07	3.0E-01	0.07	3.0E-01	96	PMID: 24097068
**Diastolic blood pressure**	0.006	0.484	0.02	0.311	-0.005	0.409	-0.01	2.9E-01	-0.01	3.0E-01	107	PMID: 27618452
**Systolic blood pressure**	0.004	0.491	0.00	0.890	0.003	0.707	-0.01	2.4E-01	-0.01	2.2E-01	86	PMID: 27618452
**GGT**	0.001	0.608	0.01	0.121	-0.020	0.143	0.00	2.7E-01	0.00	2.5E-01	25	PMID: 22001757
**CRP**	-0.037	0.631	0.01	0.939	-0.006	0.695	-0.04	5.4E-01	-0.04	5.5E-01	13	PMID: 21300955
**Insulin resistance**	0.067	0.652	0.66	0.131	-0.012	0.149	0.17	4.0E-01	0.32	1.2E-01	52	PMID: 27841877
**Adiponectin**	-0.012	0.709	0.03	0.927	-0.016	0.875	-0.01	8.4E-01	-0.01	8.5E-01	4	PMID: 23835345
**ALP**	0.000	0.841	0.00	0.498	0.008	0.492	0.00	7.1E-01	0.00	7.4E-01	14	PMID: 22001757
**Triglycerides**	0.001	0.994	-0.10	0.314	0.006	0.170	-0.09	2.9E-01	-0.09	3.0E-01	57	PMID: 24097068

Table S11. Trait associations previously reported in GWAS Catalog.

SNP	Nearest Gene	Associations of SNPs in Genomic Loci - GWAS Catalogue
**rs1800562**	*HFE*	a1c measurement, alcohol drinking, diastolic blood pressure, erythrocyte count, ferritin measurement, haematocrit, haemoglobin measurement, hepcidin:ferritin ratio, iron biomarker measurement, low density lipoprotein cholesterol measurement, mean corpuscular haemoglobin, mean corpuscular volume, serum hepcidin measurement, serum iron measurement, total cholesterol measurement, total iron binding capacity, transferrin measurement, transferrin saturation measurement
**rs1799945**	*HFE*	diastolic blood pressure, ferritin measurement, haematocrit, haemoglobin measurement, hypertension, mean arterial pressure, mean corpuscular haemoglobin, mean corpuscular haemoglobin concentration, platelet count, red blood cell distribution width, reticulocyte count, serum iron measurement, smoking status measurement, systolic blood pressure, transferrin saturation measurement
**rs855791**	*TMPRSS6*	a1c measurement, bilirubin measurement, blood protein measurement, clinical laboratory measurement, ferritin measurement, haematocrit, haemoglobin measurement, hepcidin:ferritin ratio, hepcidin:transferrin saturation ratio, iron biomarker measurement, low density lipoprotein cholesterol measurement, mean corpuscular haemoglobin, mean corpuscular haemoglobin concentration, mean corpuscular volume, red blood cell distribution width, serum hepcidin measurement, serum iron measurement, soluble transferrin receptor measurement, transferrin measurement, transferrin saturation measurement
