# Supplementary material for: Genetic studies of abdominal MRI data identify genes regulating hepcidin as major determinants of liver iron concentration
Source: J Hepatol. 2019 Sep;71(3):594–602. doi: 10.1016/j.jhep.2019.05.032 (PMC6694204; doi:10.1016/j.jhep.2019.05.032)
Supplement: Supplementary Data 2 [file CTAT_table.pdf]

CTAT methods

Tables for a "Complete, Transparent, Accurate and Timely account" (CTAT) are now mandatory for all revised submissions. The aim is to enhance the reproducibility of methods.

- Only include the parts relevant to your study
- Refer to the CTAT in the main text as 'Supplementary CTAT Table'
- Do not add subheadings
- Add as many rows as needed to include all information
- Only include one item per row

If the CTAT form is not relevant to your study, please outline the reasons why:

All methods have been described in manuscript – all generated data (liver iron data for UK Biobank participants), GWAS summary statistics) will be sent back to UK Biobank and made publicly available within six months of publication.

1.1 Antibodies

Name	Citation	Supplier	Cat no.	Clone no.

1.2 Cell lines

Name	Citation	Supplier	Cat no.	Passage no.	Authentication test method

1.3 Organisms

Name	Citation	Supplier	Strain	Sex	Age	Overall n number

1.4 Sequence based reagents

Name	Sequence	Supplier

1.5 Biological samples

Description	Source	Identifier

1.6 Deposited data

Name of repository	Identifier	Link
NA		

1.7 Software

Software name	Manufacturer	Version
GEMMA	Zhou lab	0.92
PLINK	Purcell lab	1.9

1.8 Other (*e.g.* drugs, proteins, vectors etc.)

1.9 Please provide the details of the corresponding methods author for the manuscript:

Hanieh Yaghootkar

2.0 Please confirm for randomised controlled trials all versions of the clinical protocol are included in the submission. These will be published online as supplementary information.

